# Elevated blood urea nitrogen-to-albumin ratio is associated with incident sepsis-associated delirium: evidence from a weighted competing-risk analysis in a multicenter cohort study

**DOI:** 10.3389/fnut.2026.1919287

**Published:** 2026-09-25

**Authors:** Yun Xia, Jun Jin, Jiang-Tao Deng

**Affiliations:** 1 Department of Anesthesiology, Zhongnan Hospital of Wuhan University, Wuhan, Hubei, China; 2 Department of Critical Care Medicine, The University of Hong Kong-Shenzhen Hospital, Shenzhen, Guangdong, China

**Keywords:** blood urea nitrogen-to-albumin ratio, competing-risk analysis, eICU-CRD, MIMIC-IV, risk stratification, sepsis-associated delirium

## Abstract

**Background:**

Sepsis-associated delirium (SAD) is a frequent manifestation of acute brain dysfunction in sepsis. Although the blood urea nitrogen-to-albumin ratio (BAR) has been associated with prognosis in critically ill patients, its relationship with incident SAD remains uncertain. We evaluated the association between early BAR and incident SAD.

**Methods:**

This multicenter retrospective cohort study included patients with sepsis from MIMIC-IV as the primary cohort and eICU-CRD as an independent replication cohort. BAR was calculated from eligible blood urea nitrogen and serum albumin measurements obtained within 24 h after the index time. The primary outcome was 7-day incident delirium; 3-day delirium was additionally evaluated in MIMIC-IV. Death before delirium was treated as a competing event. Covariates were selected using Boruta and LASSO regression. CBPS-based inverse probability weighting and Fine–Gray competing-risk regression were applied, with restricted cubic spline, subgroup, and exploratory XGBoost/SHAP analyses.

**Results:**

A total of 10,565 patients were included (9,612 MIMIC-IV; 953 eICU-CRD). In MIMIC-IV, the highest BAR quartile was associated with a greater subdistribution hazard of 7-day delirium vs. the lowest quartile (fully adjusted sHR 1.36, 95% CI 1.13–1.64; *P* = 0.001; *P* for trend = 0.002). Continuous BAR was also associated with 7-day delirium (sHR per 1-unit increase 1.00, 95% CI 1.00–1.01; *P* = 0.013), with similar findings for 3-day delirium. In eICU-CRD, the highest BAR quartile was likewise associated with 7-day delirium (sHR 2.20, 95% CI 1.13–4.29; *P* = 0.020; *P* for trend = 0.012), whereas the continuous association was not statistically significant (sHR 1.01, 95% CI 1.00–1.02; *P* = 0.075). Restricted cubic spline analyses suggested potential nonlinearity, particularly in MIMIC-IV. Exploratory SHAP analyses ranked BAR among the most influential model-based predictors in both cohorts.

**Conclusion:**

Higher early BAR, particularly values in the highest quartile, was associated with a greater subdistribution hazard of incident SAD, and the highest-quartile association was independently reproduced in eICU-CRD. BAR may serve as a simple risk-associated marker for identifying patients with sepsis at higher risk of incident delirium.

## Introduction

Sepsis remains a leading cause of morbidity and mortality among critically ill patients (1, 2), with systemic inflammation and organ dysfunction extending to the central nervous system (3, 4). Sepsis-associated delirium (SAD), a common manifestation of acute brain dysfunction during sepsis (5), is associated with prolonged mechanical ventilation, longer intensive care unit (ICU) and hospital stays, increased mortality, persistent cognitive impairment, and a greater healthcare burden (6, 7). Despite its clinical importance, early identification of septic patients at high risk for delirium remains challenging because SAD is a multifactorial syndrome involving systemic inflammation, metabolic stress, organ dysfunction, and impaired cerebral homeostasis (3, 8).

Previous studies have shown that malnutrition, hypercatabolism, and metabolic disturbances are closely associated with delirium in critically ill patients (9–11). In patients with sepsis, poor nutritional status has also been linked to a higher risk of SAD (12). These nutrition- and metabolism-related abnormalities may increase susceptibility to acute brain dysfunction through impaired cerebral energy metabolism, altered neurotransmitter synthesis, mitochondrial dysfunction, oxidative stress, immune dysregulation, and enhanced neuroinflammatory signaling (13–16). Inflammation may further worsen nutritional depletion by promoting endothelial leakage, albumin redistribution, and catabolic stress (17–19), thereby amplifying systemic disturbances that increase susceptibility to delirium. Elevated blood urea nitrogen (BUN) may reflect renal hypoperfusion (20), impaired nitrogenous waste clearance (21), and increased protein catabolism (22), indicating a greater systemic metabolic burden (23) that may predispose septic patients to acute brain dysfunction. Therefore, simple biomarkers that integrate nutrition–inflammation and renal–metabolic stress may help improve early SAD risk stratification.

The blood urea nitrogen-to-albumin ratio (BAR) is a readily available composite index derived from two routine laboratory parameters. Albumin reflects not only nutritional reserve but also systemic inflammation, endothelial leakage, hepatic synthetic function, and physiological reserve, whereas BUN reflects catabolic stress, renal hypoperfusion, impaired nitrogenous waste clearance, and metabolic burden (17, 24–26). Accordingly, BAR may capture a broader systemic vulnerability phenotype than either BUN or albumin alone. Previous studies have shown that elevated BAR is associated with adverse outcomes in sepsis, septic acute kidney injury, pneumonia, and other critical illnesses (27–30). However, these studies have focused mainly on mortality or general adverse outcomes, and the association between BAR and incident SAD remains insufficiently explored.

Methodological challenges should also be considered when evaluating delirium in critically ill septic patients. Patients with different BAR levels often differ substantially in illness severity, comorbidities, organ dysfunction, treatment exposures, and laboratory abnormalities, leading to baseline imbalance. Moreover, death before delirium onset can preclude subsequent delirium assessment and should be treated as a competing event rather than ignored in conventional analyses. Whether early BAR is independently associated with incident SAD after accounting for these methodological issues, and whether this association can be reproduced in an independent ICU database, remains unclear.

Therefore, we conducted a retrospective cohort study using two independent critical care databases, with the Medical Information Mart for Intensive Care IV (MIMIC-IV) as the primary cohort and the eICU Collaborative Research Database (eICU-CRD) as the independent replication cohort. We aimed to evaluate the association between BAR measured within 24 h after the index time and incident delirium within 7 days after the index time, with death treated as a competing event. To characterize this association, we applied covariate balancing propensity score (CBPS)-based inverse probability weighting, multivariable Fine–Gray competing-risk regression, restricted cubic spline analysis, subgroup analysis, and exploratory interpretable machine-learning analyses. We hypothesized that elevated BAR would be associated with a higher risk of incident SAD and may serve as a simple, low-cost risk-associated marker for early delirium risk stratification in septic ICU patients.

## Materials and methods

### Ethics statement

This multicenter retrospective cohort study was conducted using two publicly accessible critical care databases: MIMIC-IV, version 3.1, which contains data from Beth Israel Deaconess Medical Center from 2008 to 2022, and eICU-CRD, version 2.0, which includes ICU data collected from multiple hospitals across the United States during 2014–2015. Because all patient-level information in both databases had been fully de-identified, additional ethical approval and individual informed consent were not required. Database access was granted to the investigator, Deng, after successful completion of the Collaborative Institutional Training Initiative training program (certification number: 61,424,658).

### Study population

Patients with sepsis were identified using database-specific algorithms. In the MIMIC-IV cohort, sepsis was identified based on the database-derived Sepsis-3 table, and the sepsis onset time recorded in this table was used as the index time. In the eICU-CRD cohort, sepsis was identified using an operational definition based on a primary diagnosis of sepsis or septic shock combined with a Sequential Organ Failure Assessment (SOFA) score of ≥2. The earliest recorded offset of a primary sepsis or septic shock diagnosis was used to define the sepsis index time; when this diagnosis offset preceded ICU admission, the index time was set to ICU admission. Delirium was ascertained using structured bedside nursing assessment records available in each database. In MIMIC-IV, delirium status was derived from delirium-related records in the chartevents table, including CAM-ICU-related assessments and the Delirium Assessment item. Positive assessable records were classified as delirium-positive, whereas records coded as NULL, UTA (unable to assess), or otherwise non-assessable were not classified as delirium-negative. In eICU-CRD, delirium status was derived from delirium-related nursing assessment records in the nursecharting table, including categorical assessments such as Symptoms of Delirium Present and numerical delirium scale/score records. Categorical records indicating delirium were classified as positive, and numerical delirium scores ≥4 were classified as delirium-positive. When multiple assessable delirium assessments were available, the first positive assessable assessment and its timestamp were used to define delirium onset. Patients without any assessable delirium record during the relevant follow-up period were excluded. The primary outcome was incident delirium occurring within 7 days after the cohort-specific index time, whereas death before delirium onset was treated as a competing event in the time-to-event analysis. Patients were excluded if they met any of the following criteria: (1) age < 18 years; (2) repeat ICU admissions, with only the first ICU admission retained; (3) delirium occurring before the index time; (4) unavailable or unevaluable delirium assessments; (5) missing laboratory data required to calculate BAR; and (6) inability to establish a clear temporal sequence in which the BUN and albumin measurements used to calculate BAR preceded delirium onset. To preserve the temporal ordering between exposure and outcome, only BUN and albumin measurements obtained before delirium onset were eligible to contribute to BAR calculation.

A total of 41,295 patients diagnosed with sepsis were initially identified from the MIMIC-IV database (Figure 1A). After excluding 9,385 repeat ICU admissions, with no patients excluded because of age < 18 years, 31,910 patients remained. Subsequently, 1,349 patients with delirium before the index time were excluded, leaving a confirmed sepsis cohort of 30,561 patients. Among them, 8,626 patients with unavailable or unevaluable delirium assessments were excluded, leaving 21,935 patients with evaluable delirium assessments. A further 8,692 patients were excluded because the laboratory data required to calculate BAR were unavailable, leaving 13,243 patients for temporal eligibility assessment. Of these, 3,631 patients were excluded because a clear BAR-before-delirium temporal sequence could not be established. The final MIMIC-IV analytic cohort therefore comprised 9,612 patients, categorized according to cohort-specific BAR quartiles as Q1 (*n* = 2,403), Q2 (*n* = 2,403), Q3 (*n* = 2,406), and Q4 (*n* = 2,400).

**Figure 1:**
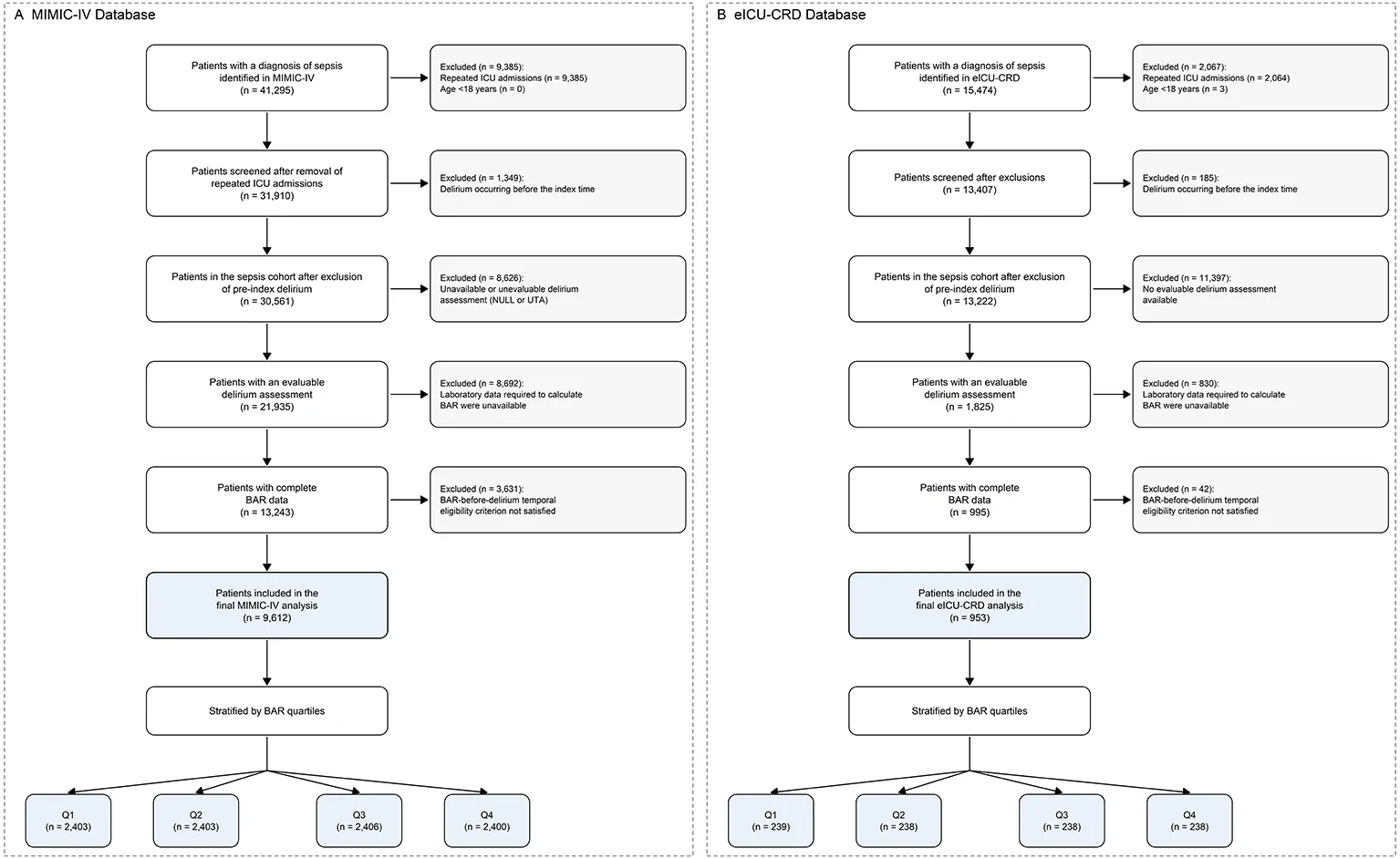
Flowchart of patient selection. **(A)** Primary cohort from the MIMIC-IV database. Patients with sepsis were initially identified (*n* = 41,295). Patients were sequentially excluded because of repeat ICU admissions (*n* = 9,385), age < 18 years (*n* = 0), delirium occurring before the index time (*n* = 1,349), unavailable or unevaluable delirium assessments recorded as NULL or UTA (*n* = 8,626), missing laboratory data required to calculate BAR (*n* = 8,692), and failure to satisfy the BAR-before-delirium temporal eligibility criterion (*n* = 3,631), yielding a final analytic cohort of 9,612 patients. **(B)** Independent replication cohort from the eICU-CRD database. Patients with sepsis were initially identified (*n* = 15,474). Patients were sequentially excluded because of repeat ICU admissions (*n* = 2,064), age < 18 years (*n* = 3), delirium occurring before the index time (*n* = 185), absence of an evaluable delirium assessment (*n* = 11,397), missing laboratory data required to calculate BAR (*n* = 830), and failure to satisfy the BAR-before-delirium temporal eligibility criterion (*n* = 42), yielding a final analytic cohort of 953 patients. Both cohorts were subsequently stratified according to cohort-specific BAR quartiles. The MIMIC-IV cohort comprised Q1 (*n* = 2,403), Q2 (*n* = 2,403), Q3 (*n* = 2,406), and Q4 (*n* = 2,400), whereas the eICU-CRD cohort comprised Q1 (*n* = 239), Q2 (*n* = 238), Q3 (*n* = 238), and Q4 (*n* = 238). BAR, blood urea nitrogen-to-albumin ratio; BUN, blood urea nitrogen; eICU-CRD, eICU Collaborative Research Database; ICU, intensive care unit; MIMIC-IV, Medical Information Mart for Intensive Care IV; NULL, never assessed; UTA, unable to assess.

Similarly, 15,474 patients diagnosed with sepsis were initially identified from the eICU-CRD database (Figure 1B). After excluding 2,064 repeat ICU admissions and three patients aged < 18 years, 13,407 patients remained. Of these, 185 patients with delirium before the index time were excluded, leaving a confirmed sepsis cohort of 13,222 patients. Subsequently, 11,397 patients without an evaluable delirium assessment were excluded, resulting in 1,825 patients with evaluable delirium assessments. A further 830 patients were excluded because the laboratory data required to calculate BAR were unavailable, leaving 995 patients for temporal eligibility assessment. Of these, 42 patients were excluded because a clear BAR-before-delirium temporal sequence could not be established. The final eICU-CRD analytic cohort therefore comprised 953 patients, categorized according to cohort-specific BAR quartiles as Q1 (*n* = 239), Q2 (*n* = 238), Q3 (*n* = 238), and Q4 (*n* = 238).

To evaluate potential selection bias, baseline characteristics were compared between patients retained in and excluded from the analysis at each of the three major selection stages: the availability of an evaluable delirium assessment, the availability of laboratory data required to calculate BAR, and fulfillment of the BAR-before-delirium temporal eligibility criterion. The corresponding comparisons are presented in Supplementary Tables S1, S2A, B for the MIMIC-IV cohort and Supplementary Tables S3, S4A, B for the eICU-CRD cohort.

### Data extraction and processing

Data were extracted from MIMIC-IV and eICU-CRD using Structured Query Language (SQL). Demographic characteristics included age, sex, and Body Mass Index (BMI). Major comorbidities included hypertension, diabetes mellitus, heart failure, Chronic Kidney Disease (CKD), dementia, and Chronic Obstructive Pulmonary Disease (COPD); malignancy was additionally assessed in the MIMIC-IV cohort. In MIMIC-IV, comorbidities were identified using International Classification of Diseases (ICD)-coded diagnoses. In eICU-CRD, comorbidities were identified from structured past-history and diagnosis records, with information from both sources combined to determine comorbidity status.

Laboratory measurements were extracted within the first 24 h after the index time. For patients who developed delirium within this 24-h window, only laboratory measurements obtained before delirium onset were eligible for analysis. Physiologically implausible values were excluded using prespecified plausible ranges incorporated into the SQL queries, and the mean of all eligible measurements was calculated for each laboratory variable. Disease severity was characterized using the SOFA score and the Glasgow Coma Scale (GCS), both of which were assessed using data obtained within 24 h of the index time. In MIMIC-IV, the Richmond Agitation-Sedation Scale (RASS) was additionally extracted within the first 24 h after the index time, and the minimum recorded value was used. Mechanical ventilation, vasoactive-agent use, and sedative exposure were extracted from structured treatment and medication records and were included as clinical covariates.

The primary exposure was BAR, calculated as the ratio of the mean BUN concentration to the mean serum albumin concentration based on eligible measurements obtained within the first 24 h after the index time. Candidate laboratory variables, depending on availability in each database, included glucose; sodium, potassium, chloride, calcium, magnesium, phosphate, bicarbonate, and anion gap; arterial pH, partial pressure of arterial oxygen (PaO_2_), partial pressure of arterial carbon dioxide (PaCO_2_), oxygen saturation, and lactate; creatinine, estimated glomerular filtration rate (eGFR), albumin, bilirubin, alanine aminotransferase (ALT), aspartate aminotransferase (AST), alkaline phosphatase, and total protein; white blood cell count (WBC), red blood cell count, hemoglobin, hematocrit, platelet count, red cell distribution width (RDW), and differential leukocyte counts; and coagulation parameters, including the international normalized ratio (INR) and partial thromboplastin time (aPTT).

During data preprocessing, candidate variables with more than 30% missing values were excluded (Supplementary Figures S1, S3). Pairwise correlations among continuous variables were assessed using Pearson correlation coefficients (Supplementary Figures S2, S4). To reduce collinearity, highly correlated variables were iteratively removed using prespecified cohort-specific correlation thresholds, with BAR retained *a priori*. Variance inflation factors (VIFs) were subsequently calculated to assess residual multicollinearity, and variables with a VIF greater than 5 were iteratively excluded.

Missing values in the retained variables were handled using multiple imputation by chained equations (MICE), implemented with the mice package in R. Five imputed datasets were generated using five MICE iterations (*m* = 5, maxit = 5; seed = 1,234). Predictive mean matching (PMM) was used for continuous variables and logistic regression for binary categorical variables. CBPS-based IPTW was implemented using the CBPS, WeightIt, and cobalt packages. Fine–Gray competing-risk analyses used the survival and cmprsk packages. LASSO and Boruta feature selection used the glmnet and Boruta packages, respectively. Machine-learning and SHAP analyses used the xgboost, SHAPforxgboost, and caret packages. All statistical analyses were performed using R software, version 4.4.1.

### Feature selection and covariate adjustment

To identify clinically relevant covariates for multivariable adjustment while reducing model complexity and redundant variable inclusion, we implemented a dual-algorithm feature-selection framework combining the Boruta algorithm and least absolute shrinkage and selection operator (LASSO) regression.

Boruta, a random forest-based wrapper algorithm, was used to identify variables with relevant predictive information by comparing the importance of each original variable with that of randomly permuted shadow variables. Only variables classified as “Confirmed” were retained. LASSO regression was subsequently performed with 10-fold cross-validation. The penalty parameter was selected based on cross-validation, and variables with non-zero coefficients at the selected penalty value were retained. Covariates identified by both Boruta and LASSO were retained for multivariable adjustment. In addition, clinically important covariates were included regardless of the feature-selection results. In MIMIC-IV, age, sex, SOFA score, and sedative exposure were additionally included; in eICU-CRD, age and sex were additionally included.

To further reduce measured confounding across BAR quartiles, CBPS was used to generate inverse probability of treatment weighting (IPTW) weights. Multicategory propensity scores were estimated for the four BAR quartile groups, targeting the average treatment effect (ATE). Covariate balance before and after weighting was evaluated using absolute standardized mean differences, with a value below 0.10 indicating adequate balance, and was visualized using Love plots.

Fine–Gray competing-risk regression models were subsequently fitted, with death before delirium onset treated as a competing event. For the quartile and trend analyses, CBPS-derived IPTW was applied, whereas continuous BAR analyses did not use quartile-derived IPTW. Model 1 was an exposure-only model; for quartile and trend analyses, this model incorporated IPTW without additional covariate adjustment. Model 2 additionally adjusted for age and sex. Model 3 further included the covariates identified by the Boruta-LASSO framework together with the prespecified clinically important covariates. Robust variance estimates were used, and associations were reported as subdistribution hazard ratios (sHRs) with corresponding 95% confidence intervals (CIs).

### Outcomes

The primary outcome was incident delirium occurring within 7 days after the index time. Incident delirium within 3 days was evaluated as a secondary outcome in the MIMIC-IV cohort. Patients with delirium occurring before the index time were excluded. Death before delirium onset within the corresponding follow-up window was treated as a competing event. For patients who did not develop delirium or experience the competing event, follow-up ended at ICU discharge or at the end of the prespecified follow-up window, whichever occurred first. Follow-up was truncated at 7 days for the primary outcome and 3 days for the secondary outcome. This competing-risk framework accounted for the possibility that death could preclude the subsequent occurrence of delirium and avoided overestimation of the cumulative incidence of delirium.

### Statistical analysis

Baseline characteristics and covariate balance. The distributions of continuous variables were assessed using the Shapiro–Wilk test. Normally distributed variables were reported as means with standard deviations, whereas non-normally distributed variables were presented as medians with interquartile ranges (IQRs). Categorical variables were summarized as frequencies and percentages. Baseline characteristics were compared across BAR quartiles using one-way analysis of variance or the Kruskal–Wallis test for continuous variables and the chi-square test for categorical variables, as appropriate. Covariate balance before and after IPTW was evaluated using absolute standardized mean differences (SMDs), with an absolute SMD < 0.10 indicating adequate balance.

#### Cumulative incidence and Fine–Gray regression

The cumulative incidence function (CIF) was used to estimate the 7-day cumulative incidence of delirium while accounting for death as a competing event. Differences among BAR quartiles were evaluated using Gray's test. CBPS was used to generate IPTW weights for the four BAR quartile groups. Weighted Fine–Gray competing-risk regression models with robust variance estimates were fitted for the quartile and trend analyses to estimate subdistribution hazard ratios (sHRs) and corresponding 95% confidence intervals (CIs). Continuous BAR analyses were performed without applying quartile-derived IPTW. Model 1 included the exposure term only; for the quartile and trend analyses, CBPS-derived IPTW was applied without additional covariate adjustment. Model 2 additionally adjusted for age and sex. Model 3 further included covariates identified by the Boruta-LASSO framework together with prespecified clinically important covariates. The first BAR quartile was used as the reference group. BAR was also analyzed as a continuous variable, and trend analyses were conducted by assigning ordinal values to the four quartiles. For analyses based on multiply imputed data, CBPS weights were re-estimated separately within each imputed dataset, the corresponding Fine–Gray models were fitted independently, and regression estimates and variances were pooled using Rubin's rules. For the secondary outcome, the corresponding Fine–Gray analyses were repeated in the MIMIC-IV cohort using incident delirium within 3 days after the index time, with death within the same period treated as the competing event.

#### Non-linear and subgroup analyses

Potential non-linear associations between continuous BAR and incident delirium were examined using restricted cubic spline (RCS) functions within the Fine–Gray competing-risk framework. Three knots were placed at the 10th, 50th, and 90th percentiles of the BAR distribution, with the median BAR value used as the reference. Overall and non-linear associations were evaluated using Wald tests. Prespecified subgroup analyses were conducted according to age (< 65 vs. ≥65 years), SOFA score (< 5 vs. ≥5), GCS score (>8 vs. ≤ 8), sex, CKD, heart failure, and COPD. Interaction terms were used to assess potential effect modification, and the results were displayed using forest plots.

#### Sensitivity and component-specific analyses

Additional sensitivity analyses were performed using cohort-specific BAR cutoffs derived from 7-day time-dependent ROC analyses by maximizing the Youden index. Cutoff-based associations were evaluated using Fine–Gray competing-risk regression with cohort-specific CBPS-derived IPTW. For multiply imputed data, weights and outcome models were re-estimated within each imputed dataset and pooled using Rubin's rules. Component-specific analyses were also performed by modeling BAR, BUN, and albumin separately within the same competing-risk framework. For quartile and trend analyses, each exposure was categorized according to its own distribution and exposure-specific CBPS-derived IPTW was estimated, whereas continuous analyses did not use quartile-derived IPTW. *E*-values were additionally calculated for the Q4-vs.-Q1 and ROC-derived cutoff comparisons to assess the potential influence of unmeasured confounding.

#### Machine-learning interpretability

As an exploratory analysis, an Extreme Gradient Boosting (XGBoost) model was fitted separately in each cohort to assess the relative importance of BAR and a harmonized set of clinical predictors for 7-day incident delirium. Shapley Additive Explanations (SHAP) values were calculated to quantify each predictor's contribution to the model output, and global SHAP summary plots were used to rank feature importance in the MIMIC-IV and eICU-CRD cohorts. Because GCS ranked among the most influential predictors in both cohorts, pairwise SHAP interaction analyses were further performed to examine the potential interaction pattern between BAR and GCS.

All statistical analyses were performed using R software, version 4.4.1 (R Foundation for Statistical Computing, Vienna, Austria). All tests were two-sided, and a *P* value < 0.05 was considered statistically significant.

## Results

### Study population and data preprocessing

A detailed flowchart of cohort derivation is presented in Figure 1. The final analysis included 9,612 patients in the MIMIC-IV primary cohort and 953 patients in the eICU-CRD independent replication cohort. The MIMIC-IV cohort was categorized into BAR quartiles: Q1 (*n* = 2,403), Q2 (*n* = 2,403), Q3 (*n* = 2,406), and Q4 (*n* = 2,400). The corresponding quartile groups in eICU-CRD included 239, 238, 238, and 238 patients, respectively. During the 7-day follow-up, 4,550 incident delirium events and 339 deaths before delirium onset occurred in MIMIC-IV; the corresponding numbers in eICU-CRD were 198 and 58, respectively.

Among the 56 prespecified candidate variables in MIMIC-IV, 11 were excluded because of missingness rates exceeding 30% (Supplementary Figure S1): eGFR, cardiac troponin T, creatine kinase, oxygen saturation, BMI, red cell distribution width standard deviation, and the absolute neutrophil, lymphocyte, monocyte, eosinophil, and basophil counts. Pairwise correlation screening was subsequently performed to identify highly correlated continuous variables (Supplementary Figure S2). This led to the removal of hematocrit, BUN, AST, lymphocyte percentage, chloride, and creatinine, while BAR was retained as the primary exposure. The remaining 39 variables were further evaluated using VIFs to assess residual multicollinearity after correlation-based filtering (Supplementary Table S8). All VIFs were below 5, with a maximum value of 3.46; therefore, no additional variables were removed. Following MICE and reintegration of the outcome and follow-up variables, the complete MIMIC-IV dataset contained 41 analysis variables.

Among the 25 prespecified candidate variables in eICU-CRD, lactate was the only variable excluded because its missingness rate exceeded 30% (44.1%; Supplementary Figure S3), leaving 24 variables. Pairwise correlation screening identified strong correlations between AST and ALT (*r* = 0.919) and between BUN and BAR (*r* = 0.901; Supplementary Figure S4). AST and BUN were subsequently removed, whereas BAR was retained as the primary exposure. The remaining 22 variables all had VIFs below 5, with a maximum value of 3.16 (Supplementary Table S9); therefore, no additional variables were removed. Following MICE and reintegration of the outcome-related variables, the complete eICU-CRD dataset contained 26 analysis variables.

Baseline comparisons between included and excluded patients were performed at successive stages of cohort selection to assess the representativeness of the final analytic cohorts (Supplementary Tables S1, S2A, B, S3, S4A, B). These comparisons were interpreted primarily using SMDs. In MIMIC-IV, patients retained after delirium-assessment screening had higher prevalences of COPD and CKD than excluded patients, whereas those retained after BAR completeness screening had higher SOFA scores and a lower prevalence of hypertension. At the subsequent temporal-eligibility stage, differences were generally smaller, with the largest imbalance observed for GCS. In eICU-CRD, patients retained after delirium-assessment screening had higher GCS scores. Patients retained after BAR completeness screening had higher SOFA scores and a higher prevalence of CKD, but were younger and had lower GCS scores and a lower prevalence of COPD than excluded patients. At the temporal-eligibility stage, patients retained in the final eICU-CRD cohort had lower SOFA scores and higher GCS scores than those excluded, with additional differences in several baseline characteristics. Therefore, the findings may be most applicable to ICU patients meeting the study sepsis criteria who had evaluable delirium assessments, available laboratory data required to calculate BAR, and an established temporal sequence in which the measurements used to calculate BAR preceded delirium onset.

### Baseline characteristics

The unweighted baseline characteristics of 9,612 patients in the MIMIC-IV primary cohort and 953 patients in the eICU-CRD independent replication cohort, stratified by cohort-specific BAR quartiles, are summarized in Table 1.

**Table 1:** Baseline characteristics of the MIMIC-IV and eICU-CRD cohorts according to blood urea nitrogen-to-albumin ratio quartiles.

Panel A. MIMIC-IV primary cohort						
Characteristic	Q1 (*n* = 2,403)	Q2 (*n* = 2,403)	Q3 (*n* = 2,406)	Q4 (*n* = 2,400)	*P* value	SMD
Demographics						
Age, years	59.00 [47.00, 69.00]	66.00 [55.00, 76.00]	68.00 [57.00, 78.00]	68.00 [57.00, 78.00]	<0.001	0.314
Male sex, *n* (%)	1,282 (53.3)	1,362 (56.7)	1,384 (57.5)	1,447 (60.3)	<0.001	0.073
Clinical severity						
SOFA score	3.00 [2.00, 4.00]	3.00 [2.00, 4.00]	3.00 [2.00, 5.00]	4.00 [3.00, 6.00]	<0.001	0.416
GCS score	15.00 [14.00, 15.00]	15.00 [14.00, 15.00]	15.00 [14.00, 15.00]	15.00 [13.00, 15.00]	<0.001	0.064
RASS	−2.00 [−4.00, 0.00]	−1.00 [−4.00, 0.00]	−1.00 [−4.00, 0.00]	−1.00 [−4.00, 0.00]	<0.001	0.101
Exposure						
BAR	3.46 [2.73, 4.13]	6.24 [5.46, 7.07]	10.49 [9.21, 12.08]	20.88 [16.80, 27.76]	<0.001	3.063
Laboratory findings						
Glucose, mg/dL	127.25 [109.33, 155.21]	134.20 [110.67, 169.00]	137.67 [110.50, 183.00]	135.00 [108.00, 182.08]	<0.001	0.150
Potassium, mmol/L	3.97 [3.65, 4.28]	4.12 [3.80, 4.50]	4.30 [3.88, 4.77]	4.53 [4.00, 5.14]	<0.001	0.487
Sodium, mmol/L	138.33 [135.67, 140.75]	138.00 [135.25, 140.50]	137.50 [134.00, 141.00]	137.00 [133.00, 140.76]	<0.001	0.053
Calcium, mg/dL	8.35 [7.82, 8.85]	8.22 [7.80, 8.75]	8.23 [7.70, 8.77]	8.18 [7.60, 8.70]	<0.001	0.093
Magnesium, mg/dL	1.90 [1.75, 2.10]	1.90 [1.73, 2.10]	2.00 [1.80, 2.20]	2.12 [1.90, 2.40]	<0.001	0.383
Phosphate, mg/dL	3.10 [2.57, 3.70]	3.30 [2.70, 3.97]	3.70 [2.96, 4.50]	4.70 [3.70, 5.98]	<0.001	0.721
Anion gap, mmol/L	13.67 [12.00, 16.00]	14.50 [12.50, 16.67]	15.50 [13.00, 18.13]	17.40 [14.60, 21.00]	<0.001	0.503
Arterial pH	7.33 [7.22, 7.39]	7.33 [7.23, 7.39]	7.33 [7.25, 7.39]	7.32 [7.24, 7.38]	0.057	0.075
PaO_2_, mmHg	96.33 [55.00, 182.34]	82.00 [48.00, 137.79]	76.00 [46.62, 118.30]	70.00 [45.00, 108.07]	<0.001	0.321
PaCO_2_, mmHg	41.33 [36.94, 47.00]	41.40 [36.67, 47.67]	40.50 [35.50, 47.00]	39.00 [33.00, 45.50]	<0.001	0.164
Lactate, mmol/L	1.87 [1.40, 2.60]	2.00 [1.47, 2.88]	2.17 [1.55, 3.26]	2.12 [1.47, 3.15]	<0.001	0.172
Albumin, g/dL	3.60 [3.10, 4.00]	3.27 [2.80, 3.70]	3.10 [2.65, 3.50]	2.80 [2.40, 3.30]	<0.001	0.583
Total bilirubin, mg/dL	0.60 [0.40, 1.30]	0.75 [0.41, 1.55]	0.75 [0.45, 1.75]	0.80 [0.40, 2.20]	<0.001	0.164
ALT, U/L	26.00 [16.50, 50.00]	29.00 [17.00, 70.00]	29.50 [17.00, 72.00]	31.42 [17.00, 75.00]	<0.001	0.080
Alkaline phosphatase, U/L	81.00 [61.00, 117.50]	86.00 [62.00, 126.25]	92.00 [66.00, 139.00]	102.00 [72.00, 163.00]	<0.001	0.156
WBC, × 10?/L	10.80 [7.50, 14.50]	11.55 [8.00, 16.32]	12.10 [8.14, 16.94]	12.80 [8.60, 18.63]	<0.001	0.163
Platelet count, × 10?/L	176.00 [127.12, 246.00]	170.00 [119.00, 241.00]	171.00 [109.05, 249.92]	168.37 [104.00, 246.62]	<0.001	0.038
Hemoglobin, g/dL	11.10 [9.60, 12.55]	10.70 [9.22, 12.23]	10.20 [8.70, 11.77]	9.33 [8.10, 11.04]	<0.001	0.369
RDW, %	14.10 [13.20, 15.67]	14.76 [13.63, 16.44]	15.30 [14.10, 17.17]	15.98 [14.65, 18.00]	<0.001	0.392
Neutrophils, %	80.40 [72.48, 86.50]	81.85 [73.40, 88.00]	82.00 [73.50, 87.90]	83.20 [76.00, 88.58]	<0.001	0.099
INR	1.25 [1.13, 1.45]	1.30 [1.20, 1.60]	1.40 [1.20, 1.75]	1.45 [1.20, 1.95]	<0.001	0.248
aPTT, s	31.05 [27.60, 36.90]	31.70 [28.00, 38.85]	32.56 [28.26, 41.09]	32.78 [28.00, 42.80]	<0.001	0.127
Comorbidities						
Hypertension, *n* (%)	1,027 (42.7)	1,009 (42.0)	823 (34.2)	635 (26.5)	<0.001	0.200
Diabetes mellitus, *n* (%)	508 (21.1)	663 (27.6)	912 (37.9)	993 (41.4)	<0.001	0.260
COPD, *n* (%)	191 (7.9)	232 (9.7)	286 (11.9)	270 (11.2)	<0.001	0.075
CKD, *n* (%)	104 (4.3)	333 (13.9)	693 (28.8)	951 (39.6)	<0.001	0.531
Heart failure, *n* (%)	363 (15.1)	667 (27.8)	857 (35.6)	940 (39.2)	<0.001	0.308
Malignancy, *n* (%)	337 (14.0)	439 (18.3)	422 (17.5)	477 (19.9)	<0.001	0.081
Dementia, *n* (%)	88 (3.7)	140 (5.8)	176 (7.3)	176 (7.3)	<0.001	0.091
Treatments						
Mechanical ventilation, *n* (%)	1,149 (47.8)	1,019 (42.4)	928 (38.6)	823 (34.3)	<0.001	0.151
Vasoactive-agent use, *n* (%)	852 (35.5)	994 (41.4)	985 (40.9)	1,049 (43.7)	<0.001	0.086
Sedative exposure, *n* (%)	1,389 (57.8)	1,224 (50.9)	1,115 (46.3)	1,005 (41.9)	<0.001	0.176
**Panel B. eICU-CRD independent replication cohort**						
**Characteristic**	**Q1 (*****n*** = **239)**	**Q2 (*****n*** = **238)**	**Q3 (*****n*** = **238)**	**Q4 (*****n*** = **238)**	***P*** **value**	**SMD**
Demographics						
Age, years	60.00 [46.00, 70.50]	69.50 [58.00, 80.00]	73.00 [61.00, 82.00]	68.00 [57.00, 80.00]	<0.001	0.404
Male sex, *n* (%)	101 (42.3)	123 (51.7)	114 (47.9)	134 (56.3)	0.018	0.154
Clinical severity						
SOFA score	5.00 [3.00, 7.00]	6.00 [4.25, 9.00]	7.00 [5.00, 10.00]	8.00 [6.00, 11.00]	<0.001	0.518
GCS score	14.00 [11.50, 15.00]	14.00 [11.00, 15.00]	14.00 [10.00, 15.00]	13.00 [9.00, 15.00]	<0.001	0.183
Exposure						
BAR	5.00 [3.69, 6.13]	9.77 [8.44, 11.35]	16.30 [14.24, 18.43]	28.83 [24.10, 37.06]	<0.001	3.238
Laboratory findings						
Creatinine, mg/dL	0.80 [0.60, 1.07]	1.26 [0.94, 1.63]	1.92 [1.34, 2.91]	2.92 [1.90, 4.30]	<0.001	0.910
Hemoglobin, g/dL	10.30 [8.92, 11.70]	9.90 [8.73, 11.30]	9.96 [8.62, 11.50]	9.33 [8.30, 10.69]	<0.001	0.165
Platelet count, × 10^9^/L	178.00 [124.50, 251.50]	159.50 [110.62, 222.75]	165.00 [104.75, 224.75]	176.00 [101.25, 255.62]	0.127	0.117
WBC, × 10^9^/L	11.90 [7.55, 17.00]	13.90 [8.70, 19.34]	15.25 [9.88, 21.77]	14.10 [9.90, 21.60]	<0.001	0.202
RDW, %	14.90 [13.90, 16.55]	15.40 [14.10, 17.16]	15.80 [14.50, 17.60]	16.00 [14.90, 17.58]	<0.001	0.217
Albumin, g/dL	2.60 [2.30, 2.90]	2.50 [2.20, 2.80]	2.37 [2.00, 2.70]	2.10 [1.80, 2.40]	<0.001	0.549
ALT, U/L	26.00 [16.00, 51.00]	27.00 [18.00, 54.50]	32.00 [17.00, 86.38]	34.00 [20.00, 67.38]	0.002	0.140
Total protein, g/dL	5.70 [5.20, 6.40]	5.60 [5.10, 6.20]	5.50 [5.00, 6.10]	5.43 [5.00, 6.00]	0.003	0.188
BMI, kg/m^2^	26.60 [21.98, 32.62]	26.81 [23.37, 32.57]	26.63 [22.34, 33.17]	27.71 [22.95, 33.19]	0.606	0.061
Comorbidities						
Hypertension, *n* (%)	103 (43.1)	149 (62.6)	132 (55.5)	145 (60.9)	<0.001	0.217
Diabetes mellitus, *n* (%)	61 (25.5)	78 (32.8)	83 (34.9)	123 (51.7)	<0.001	0.284
COPD, *n* (%)	44 (18.4)	45 (18.9)	46 (19.3)	39 (16.4)	0.847	0.041
CKD, *n* (%)	67 (28.0)	108 (45.4)	151 (63.4)	197 (82.8)	<0.001	0.685
Heart failure, *n* (%)	39 (16.3)	48 (20.2)	62 (26.1)	55 (23.1)	0.061	0.132
Treatments						
Mechanical ventilation, *n* (%)	11 (4.6)	14 (5.9)	18 (7.6)	16 (6.7)	0.580	0.068
Vasoactive-agent use, *n* (%)	42 (17.6)	65 (27.3)	87 (36.6)	88 (37.0)	<0.001	0.256
Sedative exposure, *n* (%)	22 (9.2)	32 (13.4)	44 (18.5)	55 (23.1)	<0.001	0.216

Data are presented as median [interquartile range] or *n* (%). *P* values were calculated using the Kruskal–Wallis test for continuous variables and the chi-square test for categorical variables. BAR quartile cut points were cohort specific and are shown rounded to two decimal places: MIMIC-IV, Q1 ≤ 4.78, Q2 > 4.78 to ≤ 8.08, Q3 > 8.08 to ≤ 14.17, and Q4 > 14.17; eICU-CRD, Q1 ≤ 7.32, Q2 > 7.32 to ≤ 12.79, Q3 > 12.79 to ≤ 20.71, and Q4 > 20.71. SMDs summarize the magnitude of between-quartile imbalance, with values ≥0.10 indicating potentially meaningful differences. ALT, alanine aminotransferase; aPTT, activated partial thromboplastin time; BAR, blood urea nitrogen-to-albumin ratio; BMI, body mass index; CKD, chronic kidney disease; COPD, chronic obstructive pulmonary disease; eICU-CRD, eICU Collaborative Research Database; GCS, Glasgow Coma Scale; INR, international normalized ratio; MIMIC-IV, Medical Information Mart for Intensive Care IV; PaCO_2_, partial pressure of arterial carbon dioxide; PaO_2_, partial pressure of arterial oxygen; RASS, Richmond Agitation-Sedation Scale; RDW, red cell distribution width; SMD, standardized mean difference; SOFA, Sequential Organ Failure Assessment; WBC, white blood cell count.

In MIMIC-IV, patients in the higher BAR quartiles were generally older, with the median age increasing from 59.00 years in Q1 to 68.00 years in Q4 (*P* < 0.001; SMD = 0.314). The proportion of male patients increased from 53.3 to 60.3% (*P* < 0.001; SMD = 0.073). Disease severity was greater in Q4, as reflected by an increase in the median SOFA score from 3.00 to 4.00 (*P* < 0.001; SMD = 0.416). The median GCS score remained 15.00 across all quartiles, although its distribution differed significantly among the groups (*P* < 0.001; SMD = 0.064).

Distinct laboratory patterns were observed across the MIMIC-IV BAR quartiles. Compared with Q1, patients in Q4 had lower albumin (2.80 vs. 3.60 g/dL), hemoglobin (9.33 vs. 11.10 g/dL), and PaO_2_ (70.00 vs. 96.33 mmHg), as well as higher phosphate (4.70 vs. 3.10 mg/dL), anion gap (17.40 vs. 13.67 mmol/L), potassium (4.53 vs. 3.97 mmol/L), and RDW (15.98 vs. 14.10%; all *P* < 0.001). The largest between-quartile differences among laboratory variables were observed for phosphate (SMD = 0.721), albumin (SMD = 0.583), anion gap (SMD = 0.503), and potassium (SMD = 0.487).

Comorbidity burden also differed across the MIMIC-IV BAR quartiles. From Q1 to Q4, the prevalence of CKD increased from 4.3 to 39.6%, heart failure from 15.1 to 39.2%, and diabetes mellitus from 21.1 to 41.4% (all *P* < 0.001). Conversely, the prevalence of hypertension decreased from 42.7 to 26.5% (*P* < 0.001). Mechanical ventilation and sedative exposure were less frequent in Q4 than in Q1 (34.3 vs. 47.8% and 41.9 vs. 57.8%, respectively; both *P* < 0.001), whereas vasoactive-agent use showed a smaller between-quartile imbalance (SMD = 0.086).

Broadly similar patterns were observed in eICU-CRD. From Q1 to Q4, the median age increased from 60.00 to 68.00 years, the median SOFA score increased from 5.00 to 8.00, and the median GCS score decreased from 14.00 to 13.00 (all *P* < 0.001). Higher BAR quartiles were characterized by higher creatinine (2.92 vs. 0.80 mg/dL), lower albumin (2.10 vs. 2.60 g/dL), and higher prevalences of CKD (82.8 vs. 28.0%) and diabetes mellitus (51.7 vs. 25.5%; all *P* < 0.001). Vasoactive-agent use increased from 17.6 to 37.0%, while sedative exposure increased from 9.2 to 23.1% (both *P* < 0.001). In contrast, platelet count, BMI, COPD, and mechanical ventilation did not differ significantly among the BAR quartiles.

Overall, higher BAR quartiles were characterized by greater illness severity, lower albumin levels, and a higher prevalence of several comorbidities, particularly CKD and diabetes mellitus, in both cohorts. These unweighted baseline imbalances supported the use of confounding adjustment in the subsequent weighted and multivariable analyses.

### Cumulative incidence of 7-day delirium

To estimate the cumulative incidence of incident delirium while accounting for death as a competing event, CIFs were calculated across BAR quartiles in both cohorts (Figure 2). In the MIMIC-IV cohort, the cumulative incidence curves separated early during follow-up and remained progressively ordered across increasing BAR quartiles (Gray's test, *P* < 0.001; Figure 2A). At 7 days, the cumulative incidence of delirium was approximately 39.6% in Q1, 45.1% in Q2, 48.5% in Q3, and 56.1% in Q4. Patients in Q4 (BAR > 14.17) therefore had a higher cumulative incidence than those in Q1 (BAR ≤ 4.78).

**Figure 2:**
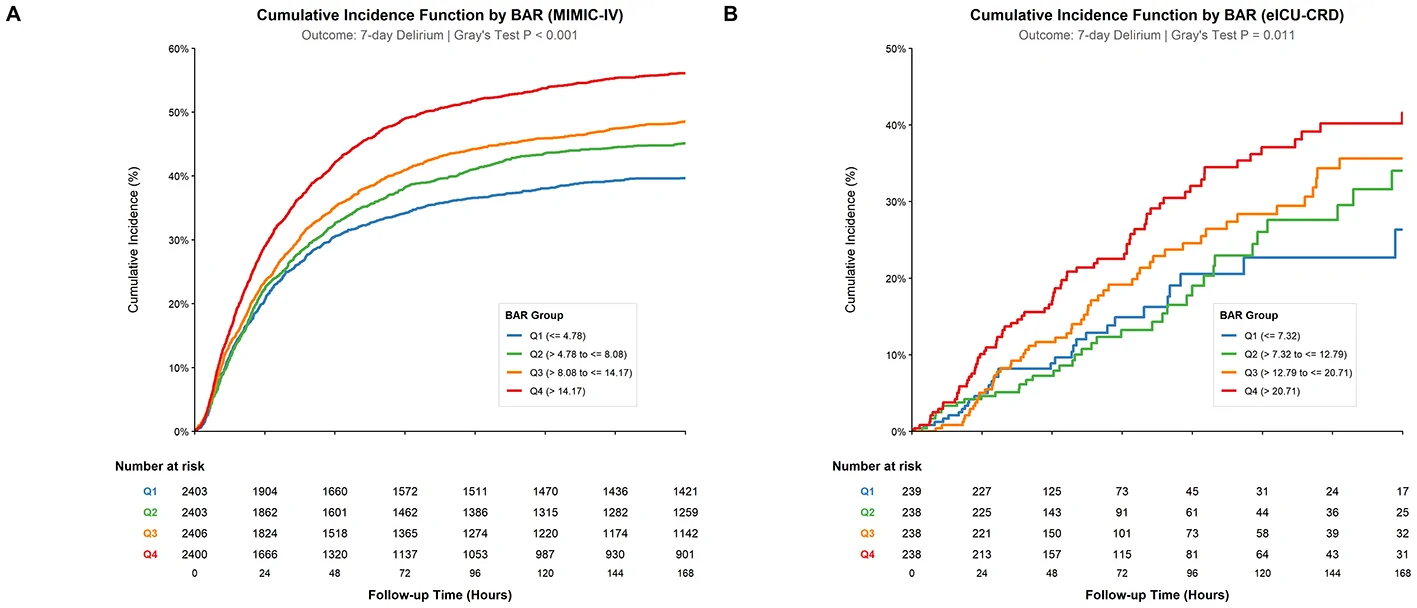
Cumulative incidence functions for incident delirium within 7 days after the index time according to cohort-specific BAR quartiles. **(A)** MIMIC-IV primary cohort. **(B)** eICU-CRD independent replication cohort. Death before delirium onset was treated as a competing event. Overall differences among the four quartiles were assessed using Gray's test. BAR, blood urea nitrogen-to-albumin ratio; CIF, cumulative incidence function; eICU-CRD, eICU Collaborative Research Database; MIMIC-IV, Medical Information Mart for Intensive Care IV.

A similar pattern was observed in the eICU-CRD cohort (Gray's test, *P* = 0.011; Figure 2B). The corresponding 7-day cumulative incidences were approximately 26.3, 34.0, 35.6, and 41.7% for Q1 through Q4, respectively. The highest BAR quartile (BAR > 20.71) exhibited the greatest cumulative incidence, whereas Q1 (BAR ≤ 7.32) had the lowest incidence. These findings indicated a consistent association between higher BAR quartiles and a greater cumulative incidence of 7-day delirium in both cohorts.

### Feature selection and covariate identification

A dual-algorithm feature-selection procedure combining LASSO regression and the Boruta algorithm was applied separately in the two cohorts. LASSO regression was performed using 10-fold cross-validation, with the penalty parameter selected based on cross-validation. In parallel, Boruta was used to identify variables with relevant importance relative to randomly generated shadow variables. Variables retained by both algorithms were carried forward for covariate adjustment, together with additional clinically selected covariates.

In the MIMIC-IV cohort, 17 variables were retained by both LASSO and Boruta: history of dementia, mechanical ventilation, RASS, COPD, GCS score, anion gap, lactate, INR, RDW, potassium, BAR, PaCO_2_, PaO_2_, total bilirubin, glucose, sodium, and vasoactive-agent use (Figure 3). BAR was retained as the primary exposure. Age, sex, SOFA score, and sedative exposure were additionally included in the fully adjusted model based on clinical relevance. The resulting Model 3 therefore included BAR as the exposure together with 20 adjustment covariates.

**Figure 3:**
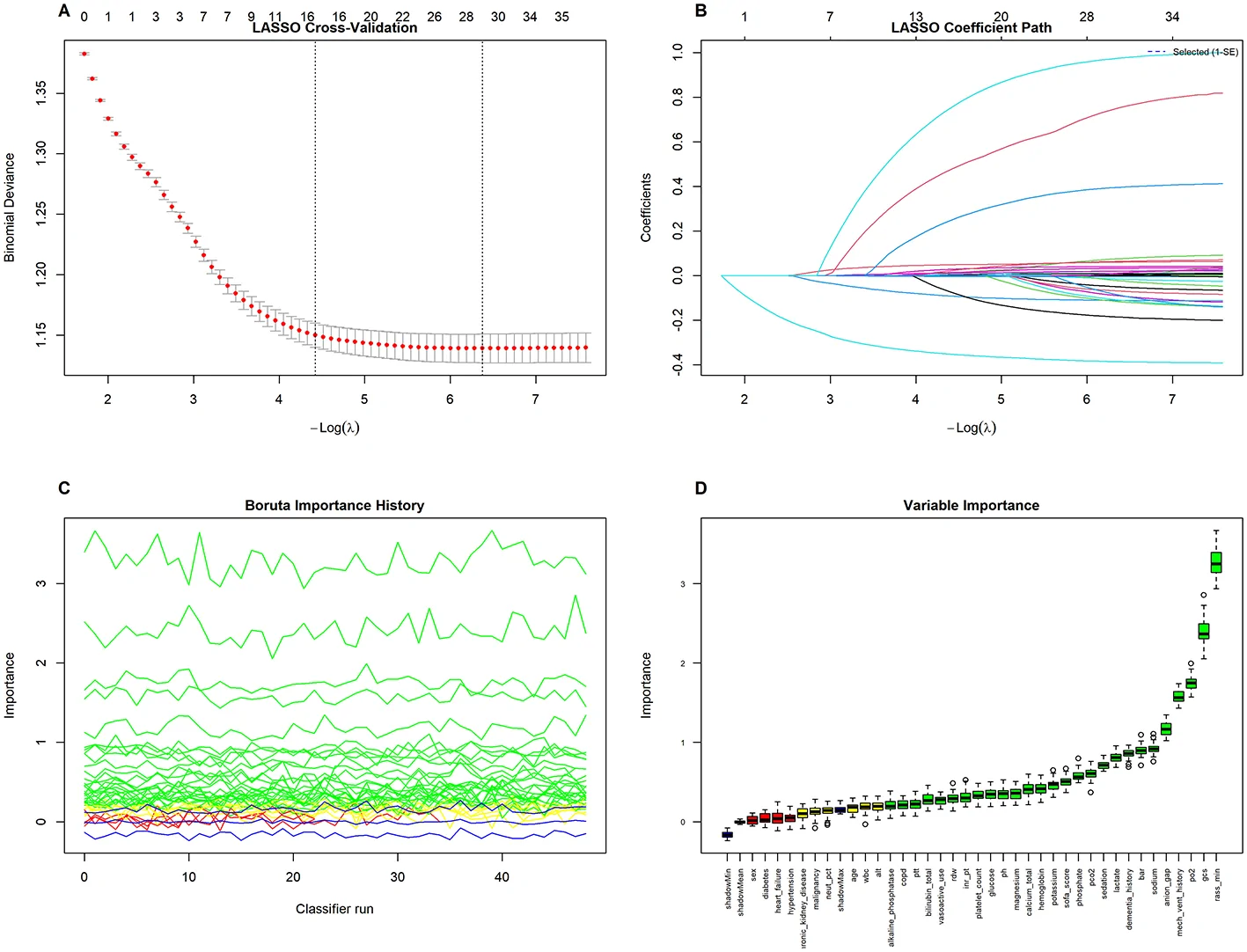
Feature selection in the MIMIC-IV primary cohort. **(A)** 10-fold cross-validation of the LASSO model. **(B)** LASSO coefficient paths. **(C)** Boruta importance history. **(D)** Boruta variable-importance distributions. Variables retained by both algorithms were carried forward for covariate adjustment, together with additional clinically selected covariates in the fully adjusted model. LASSO, least absolute shrinkage and selection operator; MIMIC-IV, Medical Information Mart for Intensive Care IV.

In the eICU-CRD cohort, six variables were retained by both algorithms: sedative exposure, GCS score, BAR, SOFA score, mechanical ventilation, and BMI (Supplementary Figure S5). BAR was retained as the primary exposure, while age and sex were additionally included in the fully adjusted model. Accordingly, the final eICU-CRD Model 3 included BAR together with age, sex, BMI, GCS score, SOFA score, sedative exposure, and mechanical ventilation.

### Dose-response association between BAR and 7-day delirium

To characterize the continuous association between BAR and incident delirium, RCS models with three knots at the 10th, 50th, and 90th percentiles were fitted within the Fine–Gray competing-risk framework, with death treated as a competing event (Figure 4). Cohort-specific median BAR values were used as the reference values, and histograms were superimposed to illustrate the distributions of BAR.

**Figure 4:**
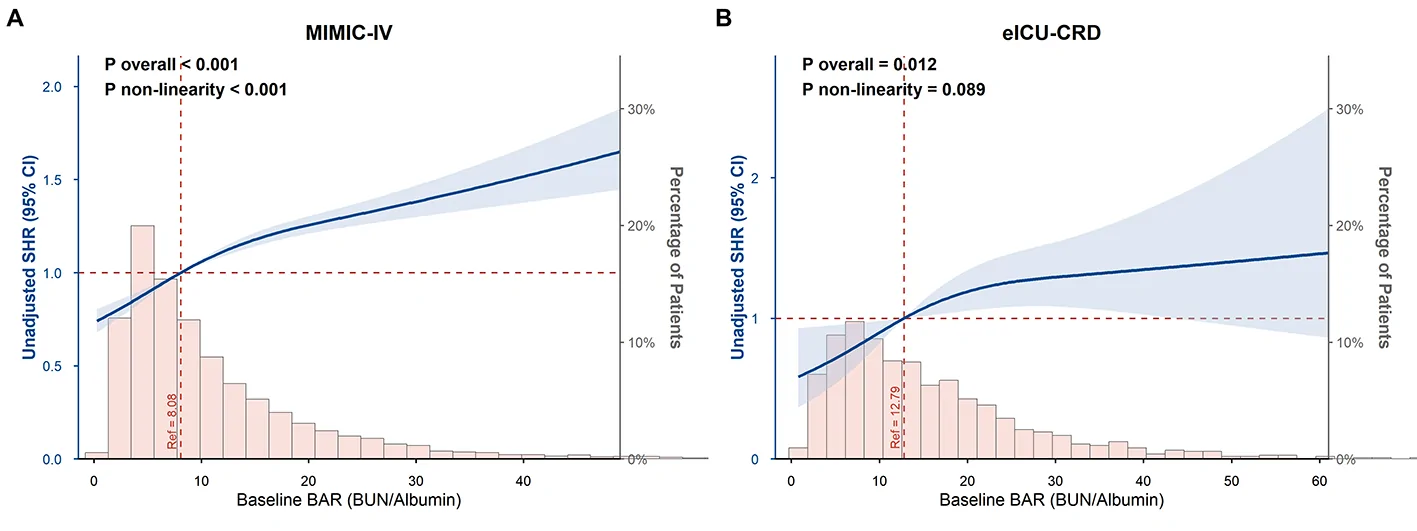
Restricted cubic spline analyses of the association between BAR and 7-day incident delirium. **(A)** MIMIC-IV primary cohort. **(B)** eICU-CRD independent replication cohort. The solid blue line represents the sHR, and the shaded area represents the 95% CI. Red dashed vertical lines indicate the cohort-specific median reference values, which were 8.08 in MIMIC-IV and 12.79 in eICU-CRD. The horizontal dashed line indicates an sHR of 1. Histograms show the distributions of BAR. Three knots were placed at the 10th, 50th, and 90th percentiles. BAR, blood urea nitrogen-to-albumin ratio; CI, confidence interval; eICU-CRD, eICU Collaborative Research Database; MIMIC-IV, Medical Information Mart for Intensive Care IV; RCS, restricted cubic spline; sHR, subdistribution hazard ratio.

In MIMIC-IV, BAR was significantly associated with the subdistribution hazard of 7-day delirium (*P* for overall association < 0.001; Figure 4A). The test for nonlinearity was also significant (*P* for nonlinearity < 0.001), indicating evidence of a nonlinear association. Using a BAR of 8.08 as the reference, the estimated sHR was below 1.0 at lower BAR values and increased above 1.0 as BAR increased. The increase was steeper across the lower BAR range and became more gradual at higher BAR values. The confidence interval remained relatively narrow across the central BAR distribution but widened toward the upper tail, where observations were less frequent.

In eICU-CRD, the overall association between BAR and 7-day delirium was also statistically significant (*P* for overall association = 0.012; Figure 4B). Using a BAR of 12.79 as the reference, the estimated sHR increased from below 1.0 at lower BAR values to above 1.0 at higher values. However, the test for nonlinearity did not reach statistical significance (*P* for nonlinearity = 0.089), indicating insufficient evidence of departure from linearity. The confidence interval widened substantially at higher BAR values, consistent with the smaller number of observations in the upper tail of the BAR distribution.

Overall, the RCS analyses showed a positive association between increasing BAR and the subdistribution hazard of 7-day delirium in both cohorts, with evidence of nonlinearity in MIMIC-IV but not in eICU-CRD.

### Covariate balance and weighting effectiveness

To reduce measured baseline confounding across the four BAR quartiles, CBPS was used to generate IPTW weights targeting the ATE estimand. Covariate balance before and after weighting was evaluated using the maximum pairwise absolute standardized mean difference (ASMD) across the four BAR quartile groups, with an ASMD < 0.10 indicating adequate balance. The corresponding Love plots are presented in Figure 5.

**Figure 5:**
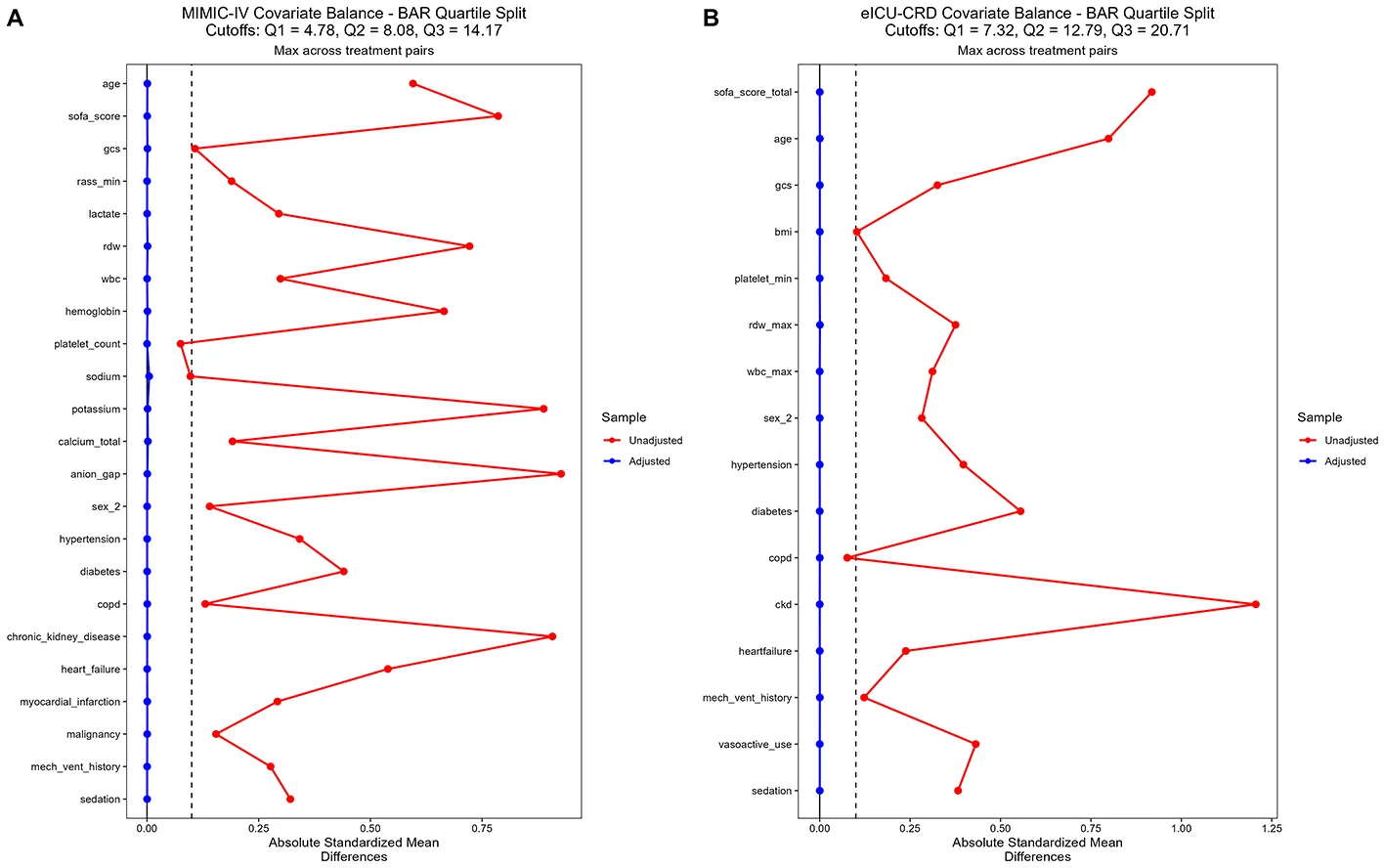
Covariate balance before and after IPTW across BAR quartiles. Love plots show the maximum pairwise ASMD across the four BAR quartile groups in the **(A)** MIMIC-IV primary cohort and **(B)** eICU-CRD independent replication cohort. Red and blue points represent covariate imbalance before and after weighting, respectively. The vertical dashed line indicates the ASMD threshold of 0.10. ASMD, absolute standardized mean difference; BAR, blood urea nitrogen-to-albumin ratio; CBPS, covariate balancing propensity score; eICU-CRD, eICU Collaborative Research Database; IPTW, inverse probability of treatment weighting; MIMIC-IV, Medical Information Mart for Intensive Care IV.

Before weighting, substantial covariate imbalance was observed in the MIMIC-IV cohort (Figure 5A). The largest imbalances were observed for anion gap, CKD, potassium, SOFA score, RDW, hemoglobin, and age. Additional differences were present across several other clinical, laboratory, comorbidity, and treatment-related variables, indicating marked heterogeneity among the unweighted BAR quartiles.

Considerable imbalance was also observed in the eICU-CRD cohort (Figure 5B). CKD showed the greatest imbalance, followed by SOFA score and age. Other notable differences involved diabetes mellitus, vasoactive-agent use, hypertension, sedative exposure, RDW, GCS score, WBC, sex, and heart failure. These findings similarly indicated substantial baseline differences across BAR quartiles before weighting.

After IPTW, covariate balance improved markedly in both cohorts. The maximum pairwise ASMDs for all variables included in the balance assessment were reduced below 0.10, indicating adequate balance of the measured covariates across the four BAR quartiles. This improvement was observed across demographic characteristics, severity measures, laboratory variables, comorbidities, and treatment-related factors.

### Association of BAR with incident delirium

Fine–Gray competing-risk regression was used to evaluate the association between BAR and incident delirium, with death before delirium onset treated as a competing event. MIMIC-IV served as the primary cohort, and eICU-CRD was used as an independent replication cohort. For BAR-quartile and trend analyses, Model 1 incorporated CBPS-derived IPTW without additional covariate adjustment, whereas continuous-BAR analyses did not use quartile-derived IPTW. Model 2 additionally adjusted for age and sex, and Model 3 further adjusted for cohort-specific covariates. The results are summarized in Table 2.

**Table 2:** Fine–Gray competing-risk regression analyses of the association between BAR and incident delirium in the MIMIC-IV and eICU-CRD cohorts.

Variable	Model 1 sHR (95% CI)	P1	Model 2 sHR (95% CI)	P_2_	Model 3 sHR (95% CI)	P3
7-day incident delirium						
MIMIC-IV primary cohort						
BAR quartiles						
Q1 (≤4.78)	1.00 (Reference)	—	1.00 (Reference)	—	1.00 (Reference)	—
Q2 (>4.78 to ≤ 8.08)	1.20 (1.01–1.43)	0.037	1.21 (1.01–1.43)	0.035	1.20 (1.01–1.42)	0.035
Q3 (>8.08 to ≤ 14.17)	1.20 (1.01–1.42)	0.041	1.20 (1.01–1.42)	0.040	1.15 (0.97–1.36)	0.100
Q4 (>14.17)	1.40 (1.15–1.70)	<0.001	1.40 (1.15–1.70)	<0.001	1.36 (1.13–1.64)	0.001
Continuous BAR						
Per 1-unit increase	1.02 (1.01–1.02)	<0.001	1.02 (1.01–1.02)	<0.001	1.00 (1.00–1.01)	0.013
P for trend	—	<0.001	—	<0.001	—	0.002
**eICU-CRD independent replication cohort**						
BAR quartiles						
Q1 (≤7.32)	1.00 (Reference)	—	1.00 (Reference)	—	1.00 (Reference)	—
Q2 (>7.32 to ≤ 12.79)	1.42 (0.71–2.83)	0.322	1.43 (0.72–2.84)	0.311	1.39 (0.71–2.73)	0.330
Q3 (>12.79 to ≤ 20.71)	1.56 (0.80–3.06)	0.193	1.58 (0.80–3.10)	0.185	1.51 (0.77–2.94)	0.232
Q4 (>20.71)	2.17 (1.06–4.44)	0.035	2.19 (1.07–4.47)	0.032	2.20 (1.13–4.29)	0.020
Continuous BAR						
Per 1-unit increase	1.01 (1.00–1.02)	0.007	1.01 (1.00–1.02)	0.015	1.01 (1.00–1.02)	0.075
*P* for trend	—	0.026	—	0.025	—	0.012
**3-day incident delirium** **MIMIC-IV cohort**						
BAR quartiles						
Q1 (≤4.51)	1.00 (Reference)	—	1.00 (Reference)	—	1.00 (Reference)	—
Q2 (>4.51 to ≤ 7.56)	1.17 (0.97–1.42)	0.102	1.17 (0.97–1.42)	0.100	1.18 (0.98–1.42)	0.083
Q3 (>7.56 to ≤ 13.46)	1.18 (0.97–1.42)	0.091	1.18 (0.98–1.42)	0.089	1.14 (0.95–1.38)	0.164
Q4 (>13.46)	1.35 (1.10–1.67)	0.005	1.35 (1.10–1.67)	0.005	1.33 (1.09–1.64)	0.006
Continuous BAR						
Per 1-unit increase	1.02 (1.01–1.02)	<0.001	1.02 (1.01–1.02)	<0.001	1.00 (1.00–1.01)	0.013
*P* for trend	—	0.005	—	0.005	—	0.009

Regression estimates were pooled across five multiply imputed datasets using Rubin's rules and are presented as sHRs with 95% CIs. Q1 was used as the reference category for BAR-quartile analyses. Death before delirium onset was treated as a competing event. For BAR-quartile and trend analyses, Fine–Gray models used CBPS-derived IPTW; continuous-BAR models did not use quartile-derived IPTW. Model 1: Included the exposure term only; for quartile and trend analyses, CBPS-derived IPTW was applied without additional covariate adjustment. Model 2: Additionally adjusted for age and sex. Model 3: Further adjusted for cohort-specific covariates. In MIMIC-IV, Model 3 included age, sex, RASS score, GCS score, INR, RDW, total bilirubin, lactate, PaCO_2_, PaO_2_, anion gap, sodium, potassium, glucose, SOFA score, history of dementia, vasoactive-agent use, mechanical ventilation, COPD, and sedative exposure. In eICU-CRD, Model 3 included age, sex, BMI, GCS score, SOFA score, sedative exposure, and mechanical ventilation. BAR, blood urea nitrogen-to-albumin ratio; BMI, body mass index; CBPS, covariate balancing propensity score; CI, confidence interval; COPD, chronic obstructive pulmonary disease; eICU-CRD, eICU Collaborative Research Database; GCS, Glasgow Coma Scale; INR, international normalized ratio; IPTW, inverse probability of treatment weighting; MIMIC-IV, Medical Information Mart for Intensive Care IV; PaCO_2_, partial pressure of arterial carbon dioxide; PaO_2_, partial pressure of arterial oxygen; RASS, Richmond Agitation-Sedation Scale; RDW, red cell distribution width; sHR, subdistribution hazard ratio; SOFA, Sequential Organ Failure Assessment.

In the MIMIC-IV cohort, continuous BAR remained associated with 7-day incident delirium after full adjustment, although the effect estimate was small (Model 3: sHR, 1.00; 95% CI, 1.00–1.01; *P* = 0.013). In the quartile-based analysis, compared with Q1, Q2 was associated with a higher subdistribution hazard of 7-day delirium (sHR, 1.20; 95% CI, 1.01–1.42; *P* = 0.035), whereas the association for Q3 did not reach statistical significance (sHR, 1.15; 95% CI, 0.97–1.36; *P* = 0.100). Q4 showed the strongest association (sHR, 1.36; 95% CI, 1.13–1.64; *P* = 0.001). The trend across BAR quartiles was statistically significant (*P* for trend = 0.002).

In the eICU-CRD cohort, the continuous association was attenuated after full adjustment and did not reach statistical significance (Model 3: sHR, 1.01; 95% CI, 1.00–1.02; *P* = 0.075). In the quartile-based analysis, Q2 (sHR, 1.39; 95% CI, 0.71–2.73; *P* = 0.330) and Q3 (sHR, 1.51; 95% CI, 0.77–2.94; *P* = 0.232) were not significantly associated with 7-day delirium, whereas Q4 was associated with a higher subdistribution hazard compared with Q1 (sHR, 2.20; 95% CI, 1.13–4.29; *P* = 0.020). The trend across BAR quartiles was significant (*P* for trend = 0.012).

The 3-day analysis in MIMIC-IV showed a broadly similar pattern. In Model 3, Q2 (sHR, 1.18; 95% CI, 0.98–1.42; *P* = 0.083) and Q3 (sHR, 1.14; 95% CI, 0.95–1.38; *P* = 0.164) were not significantly associated with 3-day incident delirium, whereas Q4 remained significantly associated with a higher subdistribution hazard (sHR, 1.33; 95% CI, 1.09–1.64; *P* = 0.006). The trend across quartiles remained significant (*P* for trend = 0.009). Continuous BAR was also associated with 3-day delirium after full adjustment (sHR per 1-unit increase, 1.00; 95% CI, 1.00–1.01; *P* = 0.013).

Overall, the quartile-based analyses showed that the highest BAR quartile was consistently associated with a higher subdistribution hazard of incident delirium in both cohorts, with significant trends across BAR quartiles. The continuous association was statistically significant in MIMIC-IV but did not reach statistical significance in the eICU-CRD cohort after full adjustment.

As an additional sensitivity analysis, Rubin-pooled Fine–Gray models using cohort-specific ROC-derived BAR cutoffs yielded similar findings. In MIMIC-IV, BAR > 6.731 was associated with a higher subdistribution hazard of 7-day delirium compared with BAR ≤ 6.731 (Model 3: sHR, 1.12; 95% CI, 1.01–1.24; *P* = 0.025). In eICU-CRD, BAR > 17.2 was likewise associated with a higher subdistribution hazard compared with BAR ≤ 17.2 (Model 3: sHR, 1.63; 95% CI, 1.19–2.24; *P* = 0.002; Supplementary Table S7).

Sensitivity analyses using *E*-values were also performed to assess the potential influence of unmeasured confounding (Supplementary Table S5). For the Q4-vs.-Q1 comparison, the *E*-values for the point estimates were 1.94 in MIMIC-IV and 3.31 in eICU-CRD, with corresponding confidence-limit *E*-values of 1.55 and 1.00, respectively. For the ROC-derived cutoff analyses, the corresponding *E*-values were 1.65 and 2.17, with confidence-limit *E*-values of 1.44 and 1.24, respectively.

### Component-specific analyses of BAR, BUN, and albumin

Component-specific Fine–Gray analyses were additionally performed to examine whether the observed association of BAR with incident delirium was similarly reflected by its individual components, BUN and albumin (Supplementary Table S6).

In MIMIC-IV, the fully adjusted Q4-vs.-Q1 association remained significant for BAR (sHR, 1.37; 95% CI, 1.14–1.66; *P* < 0.001), whereas the corresponding association for BUN was attenuated and no longer statistically significant (sHR, 1.19; 95% CI, 0.96–1.49; *P* = 0.118). Higher albumin was inversely associated with delirium, with Q4 associated with a lower subdistribution hazard compared with Q1 (sHR, 0.70; 95% CI, 0.59–0.83; *P* < 0.001).

In eICU-CRD, BAR remained associated with delirium in the Q4-vs.-Q1 comparison (sHR, 2.11; 95% CI, 1.11–4.02; *P* = 0.023), whereas neither BUN (sHR, 1.10; 95% CI, 0.54–2.25; *P* = 0.795) nor albumin (sHR, 0.90; 95% CI, 0.54–1.50; *P* = 0.679) showed significant corresponding associations (Supplementary Table S6).

### Relative importance of BAR among clinical predictors

As an exploratory analysis, XGBoost models were developed separately in the MIMIC-IV and eICU-CRD cohorts using an identical set of 13 clinical predictors to assess the relative contribution of BAR to the model output for 7-day incident delirium. SHAP values were calculated to characterize the relative importance and direction of contribution of each predictor (Figure 6).

**Figure 6:**
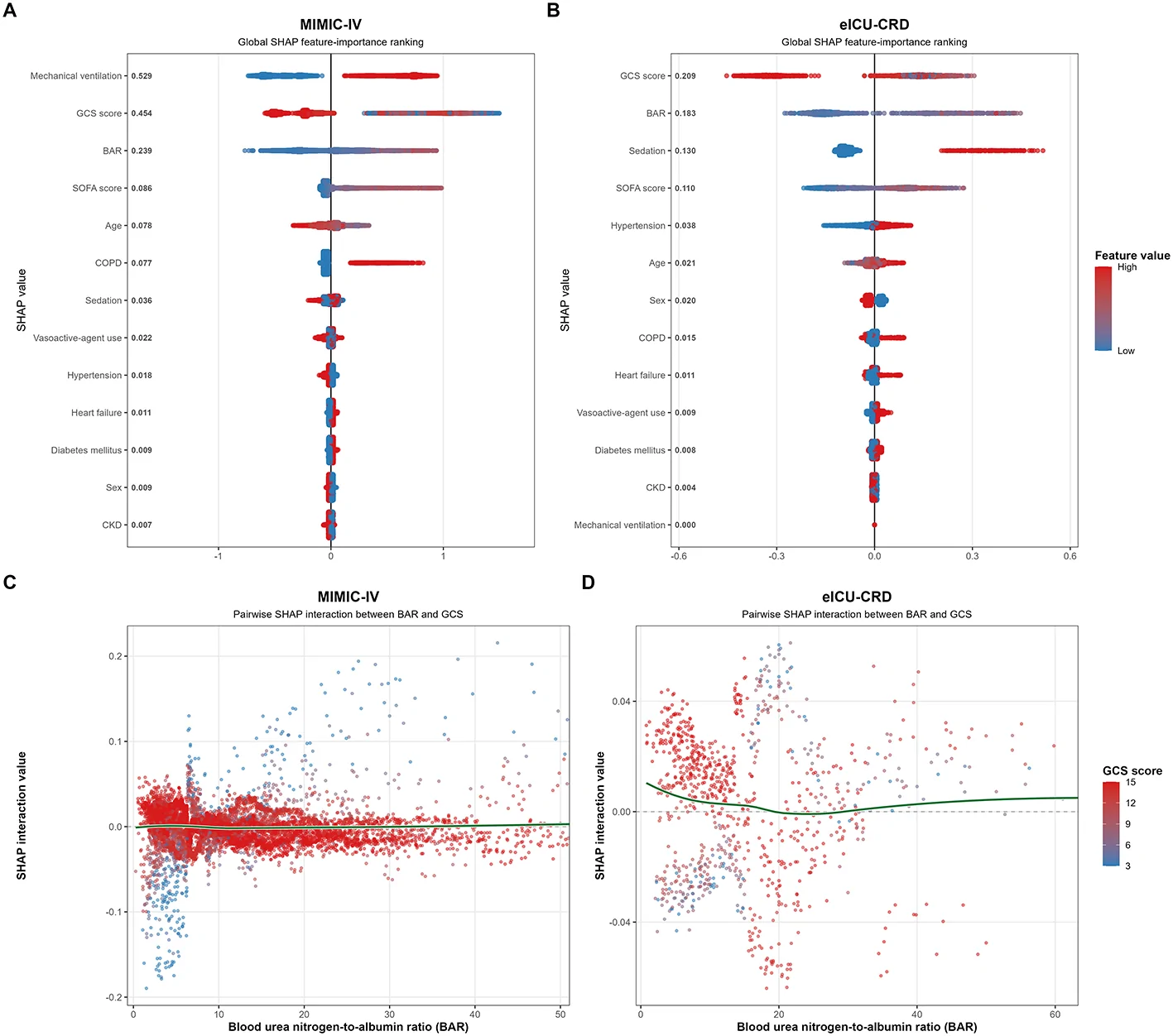
SHAP-based feature importance and BAR–GCS interactions in the MIMIC-IV and eICU-CRD cohorts. **(A)** SHAP summary plot for the MIMIC-IV cohort. **(B)** SHAP summary plot for the eICU-CRD cohort. Values indicate mean absolute SHAP values, and colors represent feature values. **(C)** Pairwise SHAP interaction between BAR and GCS in the MIMIC-IV cohort. **(D)** Pairwise SHAP interaction between BAR and GCS in the eICU-CRD cohort. Green curves show smoothed trends, and dashed horizontal lines indicate zero interaction. Positive SHAP values indicate contributions toward a higher model output for 7-day incident delirium. BAR, blood urea nitrogen-to-albumin ratio; eICU-CRD, eICU Collaborative Research Database; GCS, Glasgow Coma Scale; MIMIC-IV, Medical Information Mart for Intensive Care IV; SHAP, Shapley Additive Explanations.

In the MIMIC-IV cohort, mechanical ventilation was the highest-ranked predictor, with a mean absolute SHAP value of 0.529, followed by GCS score (0.454) and BAR (0.239; Figure 6A). BAR therefore ranked third among the 13 predictors and showed greater model-based importance than SOFA score, age, COPD, sedative exposure, and the remaining clinical variables. Higher BAR values were generally associated with positive SHAP values, whereas lower BAR values tended to be associated with negative SHAP values, indicating that higher BAR increased the model output toward 7-day delirium.

In the eICU-CRD cohort, GCS score ranked first, with a mean absolute SHAP value of 0.209, followed by BAR (0.183), sedative exposure (0.130), and SOFA score (0.110; Figure 6B). BAR therefore ranked second in the replication cohort. Higher BAR values again tended to be associated with positive SHAP contributions. Because the XGBoost models were fitted independently in the two cohorts, absolute SHAP values were interpreted within rather than directly compared between cohorts.

Because GCS ranked among the most influential predictors in both cohorts, pairwise SHAP interaction analyses were subsequently performed to explore the interaction pattern between BAR and GCS (Figures 6C, D). Interaction values were generally centered near zero in both cohorts, although localized variation was observed across different BAR and GCS levels.

### Subgroup analyses of the association between BAR and incident delirium

Subgroup analyses evaluated the association between BAR, modeled per 1-unit increase, and 7-day incident delirium across clinically relevant strata, with death treated as a competing event (Figure 7).

**Figure 7:**
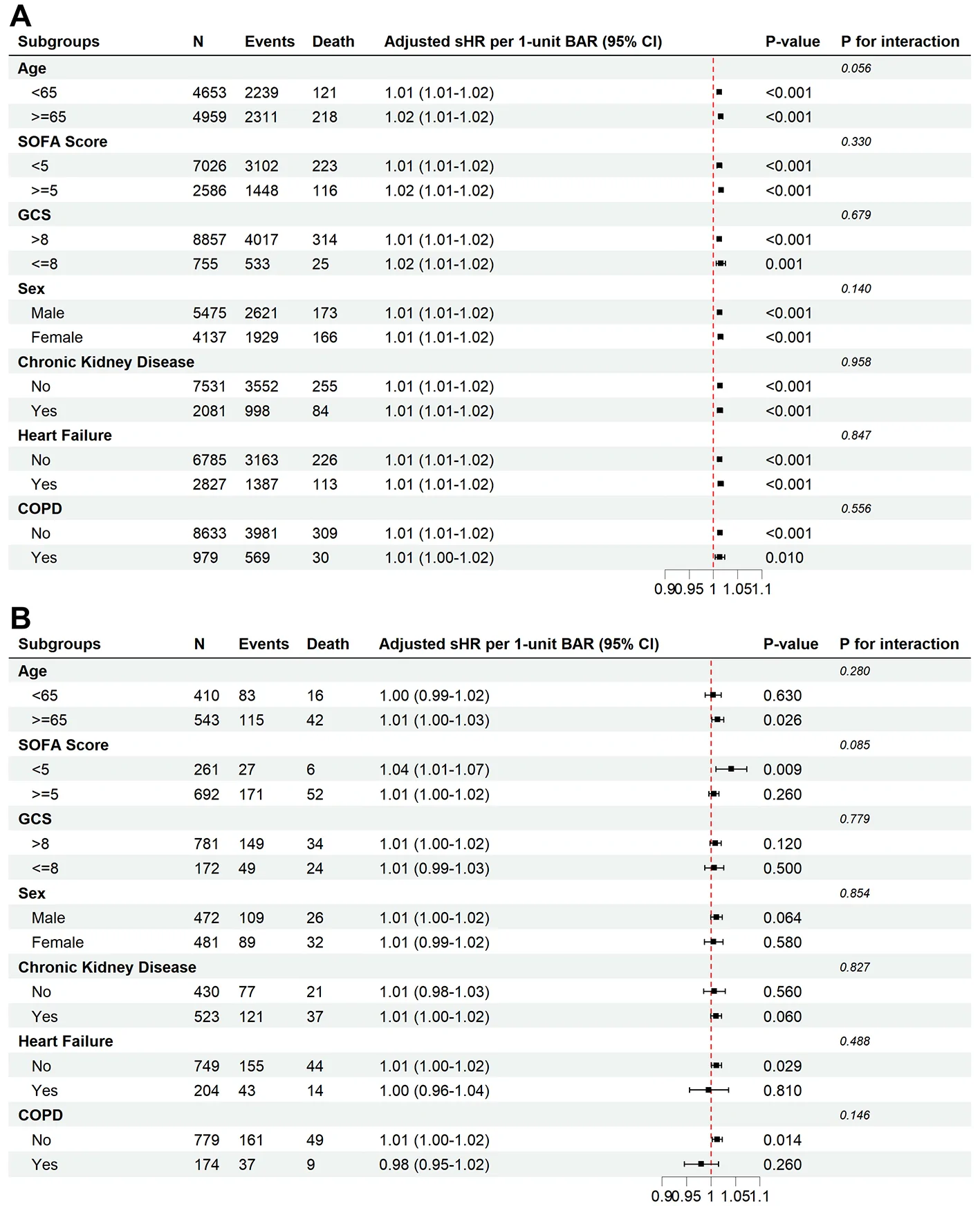
Subgroup analyses of the association between BAR and 7-day incident delirium. Adjusted sHRs and 95% CIs per 1-unit increase in BAR are presented for the **(A)** MIMIC-IV primary cohort and **(B)** eICU-CRD independent replication cohort. Squares represent point estimates, horizontal lines indicate 95% CIs, and red dashed lines denote an sHR of 1.00. BAR, blood urea nitrogen-to-albumin ratio; CI, confidence interval; CKD, chronic kidney disease; COPD, chronic obstructive pulmonary disease; eICU-CRD, eICU Collaborative Research Database; GCS, Glasgow Coma Scale; MIMIC-IV, Medical Information Mart for Intensive Care IV; sHR, subdistribution hazard ratio; SOFA, Sequential Organ Failure Assessment.

In the MIMIC-IV cohort, higher BAR was significantly associated with a greater subdistribution hazard of delirium across all examined subgroups. The estimated associations were similar across strata defined by age, SOFA score, GCS score, sex, CKD, heart failure, and COPD. No statistically significant effect modification was detected, with P values for interaction of 0.056 for age, 0.330 for SOFA score, 0.679 for GCS score, 0.140 for sex, 0.958 for CKD, 0.847 for heart failure, and 0.556 for COPD.

In the eICU-CRD cohort, significant associations were observed among patients aged ≥65 years (sHR, 1.01; 95% CI, 1.00–1.03; *P* = 0.026), those with SOFA scores < 5 (sHR, 1.04; 95% CI, 1.01–1.07; *P* = 0.009), patients without heart failure (sHR, 1.01; 95% CI, 1.00–1.02; *P* = 0.029), and patients without COPD (sHR, 1.01; 95% CI, 1.00–1.02; *P* = 0.014). However, no statistically significant interactions were detected for age (*P* for interaction = 0.280), SOFA score (*P* for interaction = 0.085), GCS score (*P* for interaction = 0.779), sex (*P* for interaction = 0.854), CKD (*P* for interaction = 0.827), heart failure (*P* for interaction = 0.488), or COPD (*P* for interaction = 0.146).

## Discussion

To our knowledge, this is the first large-scale study to specifically evaluate the association between BAR and incident sepsis-associated delirium across two independent critical care cohorts. Among 10,565 critically ill patients, including 9,612 from the MIMIC-IV primary cohort and 953 from the eICU-CRD independent replication cohort, higher BAR was associated with a greater subdistribution hazard of incident delirium after adjustment for measured confounders and accounting for death as a competing event. Methodologically, we addressed measured baseline confounding and competing risks using CBPS-derived IPTW and multivariable Fine–Gray regression, with covariate selection informed by the Boruta-LASSO framework. In the fully adjusted quartile-based analyses, patients in the highest BAR quartile had a significantly greater subdistribution hazard of 7-day incident delirium than those in the lowest quartile in both MIMIC-IV (sHR, 1.36; 95% CI, 1.13–1.64; *P* = 0.001) and eICU-CRD (sHR, 2.20; 95% CI, 1.13–4.29; *P* = 0.020), with significant trends across BAR quartiles in both cohorts. When BAR was modeled continuously, the association remained statistically significant in MIMIC-IV (sHR, 1.00; 95% CI, 1.00–1.01; *P* = 0.013) but did not reach statistical significance in eICU-CRD after full adjustment (sHR, 1.01; 95% CI, 1.00–1.02; *P* = 0.075). RCS analyses further demonstrated a positive continuous association between BAR and delirium in both cohorts, with evidence of nonlinearity in MIMIC-IV and no statistically significant evidence of departure from linearity in eICU-CRD. In exploratory XGBoost and SHAP analyses, BAR ranked among the most influential model-based predictors in both cohorts, ranking third in MIMIC-IV and second in eICU-CRD, while higher BAR values generally contributed toward a higher model output for 7-day incident delirium.

Previous studies have identified BAR as a readily available and reliable biomarker closely associated with prognosis in critically ill patients (31). In sepsis, elevated BAR has been repeatedly linked to adverse survival outcomes, including in-hospital, short-term, and long-term mortality (27, 32). Similar associations have also been reported in septic patients with acute kidney injury and in eICU-CRD-based analyses using propensity score-adjusted methods, further supporting the robustness of BAR across different critical care settings (28, 33). Beyond sepsis, BAR has been associated with poor outcomes in pneumonia, including community-acquired pneumonia and severe pneumonia, and a meta-analysis suggested that BAR may help identify pneumonia patients at high risk of ICU admission or death (29, 34, 35). Collectively, these studies indicate that BAR is not merely a mathematical combination of two routine laboratory parameters, but a clinically informative biomarker closely related to adverse outcomes in acute and critical illness. The present study extends this evidence from mortality-centered outcomes to the occurrence of acute brain dysfunction in sepsis. Unlike previous BAR studies that mainly focused on survival endpoints (36), we evaluated incident sepsis-associated delirium, a common manifestation of sepsis-associated brain dysfunction (37). From a biological perspective, this extension is plausible because BAR captures a systemic vulnerability state rather than a single organ-specific abnormality. In critically ill patients, hypoalbuminemia reflects not only impaired nutritional reserve but also inflammation-related capillary leakage and reduced physiological resilience (38, 39), whereas elevated BUN may indicate catabolic stress, renal hypoperfusion, and metabolic burden (23, 26). Poor nutritional status and hypoalbuminemia have been associated with delirium in critically ill patients, including patients with sepsis-associated delirium (9, 40). Therefore, a higher BAR may identify septic patients with a combined nutrition–inflammation and catabolic stress phenotype (41), in whom systemic illness may more readily translate into acute brain dysfunction. This interpretation is compatible with current concepts of sepsis-associated delirium as a multifactorial syndrome driven by systemic inflammation, metabolic stress, and impaired host resilience, rather than by a single neurological pathway (8).

Several biological mechanisms may plausibly contribute to the association between elevated BAR and incident sepsis-associated delirium. First, the albumin component of BAR may reflect nutritional and systemic vulnerability relevant to central nervous system dysfunction (42). In critical illness, serum albumin is influenced by nutritional status as well as systemic inflammation, increased capillary permeability, fluid status, hepatic synthetic function, and overall illness severity (43, 44). Nutritional insufficiency may increase susceptibility to delirium by impairing neurotransmitter synthesis, reducing cerebral energy metabolism, promoting mitochondrial dysfunction and oxidative stress, and altering immune regulation (45–47). Micronutrient deficiencies, including thiamine, cobalamin, and vitamin D deficiencies, have also been associated with delirium (48), potentially through neurotransmitter imbalance and enhanced inflammatory oxidative stress (16). In addition, malnutrition may disrupt intestinal barrier integrity and the gut–brain axis, thereby amplifying systemic inflammation and neuroinflammatory signaling (49). These mechanisms provide a plausible bridge between poor nutritional status, hypoalbuminemia, and acute brain dysfunction. Second, hypoalbuminemia may also reflect the inflammatory and endothelial phenotype of sepsis (24, 50). During sepsis, systemic inflammation, endothelial injury, and increased capillary permeability can promote albumin leakage from the intravascular space, leading to reduced serum albumin levels (25, 51, 52). Similar barrier dysfunction may occur at the neurovascular unit (53, 54), where disruption of the blood–brain barrier may facilitate peripheral-to-central inflammatory signaling and expose the central nervous system to circulating inflammatory mediators and plasma proteins; notably, experimental evidence suggests that albumin extravasation into the brain parenchyma may activate astrocytic TGF-β signaling, impair potassium buffering, and promote neuronal hyperexcitability, thereby disturbing cerebral homeostasis (55, 56). Therefore, low albumin may serve as an accessible peripheral marker of a broader inflammation–endothelial leakage state that is relevant to sepsis-associated brain dysfunction (51). Third, the BUN component of BAR may reflect a complementary catabolic–renal–metabolic stress pathway. Elevated BUN in sepsis may arise from increased protein catabolism, renal hypoperfusion, impaired renal clearance, neurohormonal activation, and metabolic burden (26, 57–59). These abnormalities are closely linked to acute brain dysfunction because renal dysfunction and accumulation of nitrogenous or uremic solutes can disturb cerebral metabolic homeostasis, electrolyte and acid–base balance, and neuroinflammatory responses (60, 61). Clinically, acute kidney injury is frequently accompanied by delirium in critically ill patients, and elevated BUN has been associated with delirium risk even among critically ill elderly patients without overt kidney disease (62–64). Thus, the BUN component may connect BAR with the kidney–brain axis and the metabolic encephalopathy spectrum.

Taken together, BAR may integrate several delirium-relevant systemic domains into a single readily available index. By combining low albumin, which may reflect nutritional status, inflammation, endothelial leakage, and reduced physiological reserve, with elevated BUN, which may reflect catabolic stress, renal hypoperfusion, and metabolic burden, a high BAR may capture a broader systemic vulnerability profile. In this sense, BAR may integrate nutritional–inflammatory and renal–metabolic information, thereby capturing a broader host-vulnerability phenotype than either BUN or albumin alone. BAR should therefore be interpreted not as a direct marker of a single neurological pathway, but as an integrative peripheral biomarker of susceptibility to sepsis-associated delirium. Consistent with this interpretation, component-specific analyses showed that the associations of BUN and albumin alone were less consistent across the two cohorts than those observed for BAR. These proposed mechanisms provide biological context for the observed association but should not be interpreted as causal pathways demonstrated by the present study.

### Strengths and clinical implications

This study has several strengths. First, we evaluated the association between BAR and incident sepsis-associated delirium using two independent ICU databases, with MIMIC-IV serving as the primary cohort and eICU-CRD as the independent replication cohort. This design allowed us to assess whether the observed association was reproducible across different data sources and clinical settings, thereby strengthening the credibility of our findings. Second, our analytic strategy was designed to address several methodological challenges inherent to delirium research in critically ill patients. We used the Boruta algorithm and LASSO regression to identify covariates for multivariable adjustment, applied CBPS-based IPTW to improve covariate balance across BAR quartiles, and fitted Fine–Gray competing-risk models to account for death before delirium onset. This framework allowed us to address measured baseline confounding and the competing risk of death within a unified analytical approach. Third, we examined BAR in both categorical and continuous forms and further characterized the continuous association using restricted cubic splines. The association of the highest BAR quartile with incident delirium and the significant trend across BAR quartiles were reproduced in both cohorts, while the RCS analyses further characterized the shape of the association across the BAR distribution. In addition, exploratory XGBoost and SHAP analyses characterized the relative contribution of BAR within the fitted machine-learning models. From a clinical perspective, BAR is attractive because both BUN and albumin are inexpensive, routinely measured, and widely available in ICU practice. Elevated BAR may serve as a readily available risk-associated marker for identifying septic patients at higher risk of incident delirium. However, whether BAR-based risk stratification provides clinically useful information beyond established risk factors or improves patient management and outcomes requires prospective evaluation.

### Limitations

This study has several limitations. First, owing to its retrospective observational design, causal inference cannot be established, and residual confounding remains possible despite CBPS-derived IPTW and multivariable adjustment. In particular, BAR is closely related to renal function through its BUN component, and residual confounding from AKI, azotemia, RRT, or other incompletely measured renal factors cannot be excluded. Therefore, BAR should be interpreted as a risk-associated marker rather than as a causal determinant of delirium or a marker with an effect independent of renal function.

Second, delirium ascertainment relied on routinely documented bedside ICU assessments. Assessment frequency was not uniform across patients or hospitals, and fluctuating or hypoactive delirium may therefore have been underdetected. In addition, patients without evaluable delirium assessments or sufficient laboratory data for BAR calculation were excluded, and the revised temporal eligibility criterion introduced further exclusions. These selection processes may limit generalizability, particularly in eICU-CRD, where included and excluded patients differed in several baseline characteristics. Third, BAR was calculated from eligible BUN and albumin measurements obtained within 24 h after the index time; for patients who developed delirium during this window, only measurements obtained before delirium onset were included. Although averaging eligible measurements reduced reliance on a single laboratory value, this approach reflects the early BAR level and does not capture longitudinal changes during the ICU stay. Moreover, both BUN and albumin may be influenced by fluid therapy, renal function, catabolic status, nutritional status, hepatic function, inflammation, and illness severity. Fourth, death before delirium onset was treated as a competing event, whereas ICU discharge without prior delirium marked the end of the prespecified observation period. Because discharge timing may be related to clinical recovery and illness severity, informative discharge-related censoring cannot be completely excluded. Fifth, eICU-CRD served as an independent multicenter replication cohort and was substantially smaller than the MIMIC-IV cohort, resulting in wider confidence intervals and more limited statistical precision. Potential between-center heterogeneity in case mix, treatment practices, delirium assessment, laboratory testing, and documentation may also have influenced the estimates. Finally, the biological mechanisms underlying the association between BAR and sepsis-associated delirium could not be directly examined. The proposed nutritional, inflammatory, renal, metabolic, and neurovascular pathways should therefore be regarded as biologically plausible context for the observed association rather than as confirmed causal mechanisms.

## Conclusions

In conclusion, higher early BAR, particularly values in the highest quartile, was associated with an increased subdistribution hazard of incident sepsis-associated delirium among critically ill patients with sepsis, and the highest-quartile association was independently reproduced in the eICU-CRD cohort. RCS analyses further suggested a potentially nonlinear association, particularly in MIMIC-IV. Derived from routinely available BUN and albumin measurements, BAR may serve as a simple risk-associated marker for identifying patients at higher risk of incident delirium. Further prospective studies are needed to determine whether BAR-based risk stratification provides clinically useful information beyond established risk factors or improves patient management and outcomes.

## Data Availability

Publicly available datasets were analyzed in this study. This data can be found here: MIMIC-IV: https://physionet.org/content/mimiciv/3.1/; eICU-CRD: https://physionet.org/content/eicu-crd/. Access to both de-identified datasets requires completion of human-subjects protection training and signing of a PhysioNet Credentialed Data Use Agreement.

## Supplementary material

Supplementary Figure S1 Missingness of candidate variables in the MIMIC-IV cohort.

Supplementary Figure S2 Correlation heatmap before collinearity screening in the MIMIC-IV cohort.

Supplementary Figure S3 Missingness of candidate variables in the eICU-CRD cohort.

Supplementary Figure S4 Correlation heatmap before collinearity screening in the eICU-CRD cohort.

Supplementary Figure S5 Feature selection using LASSO regression and the Boruta algorithm in the eICU-CRD independent replication cohort.

Supplementary Table S1 Baseline comparison according to delirium assessment eligibility in the MIMIC-IV cohort.

Supplementary Table S2A Baseline comparison according to BAR data completeness in the MIMIC-IV cohort.

Supplementary Table S2B Baseline comparison according to temporal eligibility in the MIMIC-IV cohort.

Supplementary Table S3 Baseline comparison according to delirium assessment eligibility in the eICU-CRD cohort.

Supplementary Table S4A Baseline comparison according to BAR data completeness in the eICU-CRD cohort.

Supplementary Table S4B Baseline comparison according to temporal eligibility in the eICU-CRD cohort.

Supplementary Table S5 *E*-value sensitivity analyses for unmeasured confounding.

Supplementary Table S6 Component-specific Fine–Gray analyses of BAR, BUN, and albumin.

Supplementary Table S7 Sensitivity analyses using cohort-specific ROC-derived BAR cutoffs.

Supplementary Table S8 Final variance inflation factors in the MIMIC-IV cohort.

Supplementary Table S9 Final variance inflation factors in the eICU-CRD cohort.
