# Supplementary material for: Elevated blood urea nitrogen-to-albumin ratio is associated with incident sepsis-associated delirium: evidence from a weighted competing-risk analysis in a multicenter cohort study
Source: Front Nutr. 2026 Sep 25;13:1919287. doi: 10.3389/fnut.2026.1919287 (PMC13649452; doi:10.3389/fnut.2026.1919287)
Supplement: Supplementary file 1 [file Table_1.docx]

| Table S1. Comparison of baseline characteristics between patients with and without an evaluable delirium assessment in the MIMIC-IV cohort | | | | | |
| --- | --- | --- | --- | --- | --- |
| **Variables** | **Overall (N = 30,561)** | **Excluded (N = 8,626)** | **Included (N = 21,935)** | **SMD** | **P-value** |
| **Demographics** |  |  |  |  |  |
| Age, years (Mean ± SD) | 66.70 ± 16.23 | 65.95 ± 16.62 | 67.00 ± 16.06 | 0.064 | <0.001 |
| Female sex, n (%) | 12,875 (42.1) | 3,600 (41.7) | 9,275 (42.3) | 0.011 | 0.381 |
| **Clinical Severity** |  |  |  |  |  |
| SOFA score (Mean ± SD) | 3.61 ± 1.98 | 3.68 ± 2.07 | 3.58 ± 1.94 | 0.049 | <0.001 |
| GCS score (Mean ± SD) | 14.31 ± 1.94 | 14.24 ± 2.14 | 14.33 ± 1.86 | 0.045 | <0.001 |
| **Comorbidities** |  |  |  |  |  |
| Diabetes Mellitus, n (%) | 9,447 (30.9) | 2,539 (29.4) | 6,908 (31.5) | 0.045 | <0.001 |
| Hypertension, n (%) | 12,526 (41.0) | 3,659 (42.4) | 8,867 (40.4) | 0.040 | 0.001 |
| Congestive Heart Failure, n (%) | 8,780 (28.7) | 2,250 (26.1) | 6,530 (29.8) | 0.082 | <0.001 |
| Malignancy, n (%) | 5,084 (16.6) | 1,301 (15.1) | 3,783 (17.2) | 0.059 | <0.001 |
| Chronic Kidney Disease, n (%) | 5,979 (19.6) | 1,407 (16.3) | 4,572 (20.8) | 0.117 | <0.001 |
| COPD, n (%) | 2,499 (8.2) | 394 (4.6) | 2,105 (9.6) | 0.197 | <0.001 |
| Abbreviations: SMD, standardized mean difference; SOFA, Sequential Organ Failure Assessment; GCS, Glasgow Coma Scale; COPD, chronic obstructive pulmonary disease. | | | | | |
