# Supplementary material for: Elevated blood urea nitrogen-to-albumin ratio is associated with incident sepsis-associated delirium: evidence from a weighted competing-risk analysis in a multicenter cohort study
Source: Front Nutr. 2026 Sep 25;13:1919287. doi: 10.3389/fnut.2026.1919287 (PMC13649452; doi:10.3389/fnut.2026.1919287)
Supplement: Supplementary file 2 [file Table_2.docx]

| Table S2A. Comparison of baseline characteristics between patients with complete BAR data and those excluded because of missing BAR values in the MIMIC-IV cohort | | | | | |
| --- | --- | --- | --- | --- | --- |
| **Variables** | **Overall (N = 21,935)** | **Excluded (N = 8,692)** | **Included (N = 13,243)** | **SMD** | **P-value** |
| **Demographics** |  |  |  |  |  |
| Age, years (Mean ± SD) | 64.94 ± 16.01 | 65.84 ± 15.86 | 64.34 ± 16.07 | 0.094 | <0.001 |
| Female sex, n (%) | 9,275 (42.3) | 3,591 (41.3) | 5,684 (42.9) | 0.033 | 0.018 |
| **Clinical Severity** |  |  |  |  |  |
| SOFA score (Mean ± SD) | 3.58 ± 1.94 | 3.29 ± 1.70 | 3.78 ± 2.06 | 0.259 | <0.001 |
| GCS score (Mean ± SD) | 13.41 ± 2.93 | 13.30 ± 3.02 | 13.47 ± 2.86 | 0.058 | <0.001 |
| **Comorbidities** |  |  |  |  |  |
| Diabetes Mellitus, n (%) | 6,908 (31.5) | 2,704 (31.1) | 4,204 (31.7) | 0.014 | 0.321 |
| Hypertension, n (%) | 8,867 (40.4) | 3,907 (44.9) | 4,960 (37.5) | 0.153 | <0.001 |
| Congestive Heart Failure, n (%) | 6,530 (29.8) | 2,566 (29.5) | 3,964 (29.9) | 0.009 | 0.515 |
| Malignancy, n (%) | 3,783 (17.2) | 1,527 (17.6) | 2,256 (17.0) | 0.014 | 0.307 |
| Chronic Kidney Disease, n (%) | 4,572 (20.8) | 1,703 (19.6) | 2,869 (21.7) | 0.051 | <0.001 |
| COPD, n (%) | 2,105 (9.6) | 782 (9.0) | 1,323 (10.0) | 0.034 | 0.015 |
| Abbreviations: SMD, standardized mean difference; SOFA, Sequential Organ Failure Assessment; GCS, Glasgow Coma Scale; COPD, chronic obstructive pulmonary disease. | | | | | |
