# Supplementary material for: Elevated blood urea nitrogen-to-albumin ratio is associated with incident sepsis-associated delirium: evidence from a weighted competing-risk analysis in a multicenter cohort study
Source: Front Nutr. 2026 Sep 25;13:1919287. doi: 10.3389/fnut.2026.1919287 (PMC13649452; doi:10.3389/fnut.2026.1919287)
Supplement: Supplementary file 3 [file Table_3.docx]

| Table S2B. Comparison of baseline characteristics between patients meeting versus not meeting the BAR-before-delirium criterion in the MIMIC-IV cohort | | | | | |
| --- | --- | --- | --- | --- | --- |
| **Variables** | **Overall (N = 13,243)** | **Excluded (N = 3,631)** | **Included (N = 9,612)** | **SMD** | **P-value** |
| **Demographics** |  |  |  |  |  |
| Age, years (Mean ± SD) | 64.34 ± 16.07 | 65.15 ± 15.94 | 64.04 ± 16.11 | 0.069 | <0.001 |
| Female sex, n (%) | 5,684 (42.9) | 1,547 (42.6) | 4,137 (43.0) | 0.009 | 0.652 |
| **Clinical Severity** |  |  |  |  |  |
| SOFA score (Mean ± SD) | 3.78 ± 2.06 | 3.63 ± 1.97 | 3.83 ± 2.09 | 0.099 | <0.001 |
| GCS score (Mean ± SD) | 13.47 ± 2.86 | 13.22 ± 3.08 | 13.57 ± 2.77 | 0.118 | <0.001 |
| **Comorbidities** |  |  |  |  |  |
| Diabetes Mellitus, n (%) | 4,204 (31.7) | 1,128 (31.1) | 3,076 (32.0) | 0.020 | 0.302 |
| Hypertension, n (%) | 4,960 (37.5) | 1,466 (40.4) | 3,494 (36.4) | 0.083 | <0.001 |
| Congestive Heart Failure, n (%) | 3,964 (29.9) | 1,137 (31.3) | 2,827 (29.4) | 0.041 | 0.033 |
| Malignancy, n (%) | 2,256 (17.0) | 581 (16.0) | 1,675 (17.4) | 0.038 | 0.052 |
| Chronic Kidney Disease, n (%) | 2,869 (21.7) | 788 (21.7) | 2,081 (21.7) | 0.001 | 0.948 |
| COPD, n (%) | 1,323 (10.0) | 344 (9.5) | 979 (10.2) | 0.024 | 0.223 |
| Abbreviations: SMD, standardized mean difference; SOFA, Sequential Organ Failure Assessment; GCS, Glasgow Coma Scale; COPD, chronic obstructive pulmonary disease. | | | | | |
