# Supplementary material for: Elevated blood urea nitrogen-to-albumin ratio is associated with incident sepsis-associated delirium: evidence from a weighted competing-risk analysis in a multicenter cohort study
Source: Front Nutr. 2026 Sep 25;13:1919287. doi: 10.3389/fnut.2026.1919287 (PMC13649452; doi:10.3389/fnut.2026.1919287)
Supplement: Supplementary file 4 [file Table_4.docx]

| Table S3. Comparison of baseline characteristics between patients with and without an evaluable delirium assessment in the eICU-CRD cohort | | | | | |
| --- | --- | --- | --- | --- | --- |
| **Variables** | **Overall (N = 13,222)** | **Excluded (N = 11,397)** | **Included (N = 1,825)** | **SMD** | **P-value** |
| **Demographics** |  |  |  |  |  |
| Age, years (Mean ± SD) | 66.24 ± 16.08 | 66.18 ± 16.01 | 66.66 ± 16.53 | 0.030 | 0.244 |
| Female sex, n (%) | 6,329 (47.9) | 5,417 (47.5) | 912 (50.0) | 0.049 | 0.054 |
| **Clinical Severity** |  |  |  |  |  |
| SOFA score (Mean ± SD) | 6.77 ± 3.82 | 6.79 ± 3.87 | 6.62 ± 3.52 | 0.047 | 0.054 |
| GCS score (Mean ± SD) | 11.47 ± 3.96 | 11.31 ± 4.04 | 12.20 ± 3.52 | 0.234 | <0.001 |
| **Comorbidities** |  |  |  |  |  |
| Diabetes Mellitus, n (%) | 4,745 (35.9) | 4,107 (36.0) | 638 (35.0) | 0.023 | 0.373 |
| Hypertension, n (%) | 6,980 (52.8) | 5,944 (52.2) | 1,036 (56.8) | 0.093 | <0.001 |
| Congestive Heart Failure, n (%) | 2,765 (20.9) | 2,347 (20.6) | 418 (22.9) | 0.056 | 0.024 |
| Chronic Kidney Disease, n (%) | 6,074 (45.9) | 5,160 (45.3) | 914 (50.1) | 0.096 | <0.001 |
| COPD, n (%) | 2,469 (18.7) | 2,100 (18.4) | 369 (20.2) | 0.045 | 0.068 |
| Abbreviations: COPD, chronic obstructive pulmonary disease; GCS, Glasgow Coma Scale; SMD, standardized mean difference; SOFA, Sequential Organ Failure Assessment. | | | | | |
