# Supplementary material for: Elevated blood urea nitrogen-to-albumin ratio is associated with incident sepsis-associated delirium: evidence from a weighted competing-risk analysis in a multicenter cohort study
Source: Front Nutr. 2026 Sep 25;13:1919287. doi: 10.3389/fnut.2026.1919287 (PMC13649452; doi:10.3389/fnut.2026.1919287)
Supplement: Supplementary file 5 [file Table_5.docx]

| Table S4A. Comparison of baseline characteristics between patients with complete BAR data and those excluded because of missing BAR values in the eICU-CRD cohort | | | | | |
| --- | --- | --- | --- | --- | --- |
| **Variables** | **Overall (N = 1,825)** | **Excluded (N = 830)** | **Included (N = 995)** | **SMD** | **P-value** |
| **Demographics** |  |  |  |  |  |
| Age, years (Mean ± SD) | 66.66 ± 16.53 | 67.98 ± 16.25 | 65.56 ± 16.69 | 0.147 | 0.002 |
| Female sex, n (%) | 912 (50.0) | 407 (49.0) | 505 (50.8) | 0.034 | 0.465 |
| **Clinical Severity** |  |  |  |  |  |
| SOFA score (Mean ± SD) | 6.62 ± 3.52 | 5.89 ± 3.07 | 7.23 ± 3.76 | 0.390 | <0.001 |
| GCS score (Mean ± SD) | 12.20 ± 3.52 | 12.42 ± 3.34 | 12.01 ± 3.67 | 0.117 | 0.019 |
| **Comorbidities** |  |  |  |  |  |
| Diabetes Mellitus, n (%) | 638 (35.0) | 281 (33.9) | 357 (35.9) | 0.042 | 0.367 |
| Hypertension, n (%) | 1,036 (56.8) | 488 (58.8) | 548 (55.1) | 0.075 | 0.11 |
| Congestive Heart Failure, n (%) | 418 (22.9) | 203 (24.5) | 215 (21.6) | 0.068 | 0.149 |
| Chronic Kidney Disease, n (%) | 914 (50.1) | 366 (44.1) | 548 (55.1) | 0.221 | <0.001 |
| COPD, n (%) | 369 (20.2) | 189 (22.8) | 180 (18.1) | 0.116 | 0.013 |
| Abbreviations: SMD, standardized mean difference; SOFA, Sequential Organ Failure Assessment; GCS, Glasgow Coma Scale; COPD, chronic obstructive pulmonary disease. | | | | | |
