# Supplementary material for: Elevated blood urea nitrogen-to-albumin ratio is associated with incident sepsis-associated delirium: evidence from a weighted competing-risk analysis in a multicenter cohort study
Source: Front Nutr. 2026 Sep 25;13:1919287. doi: 10.3389/fnut.2026.1919287 (PMC13649452; doi:10.3389/fnut.2026.1919287)
Supplement: Supplementary file 6 [file Table_6.docx]

| Table S4B. Comparison of baseline characteristics between patients meeting versus not meeting the BAR-before-delirium criterion in the eICU-CRD cohort | | | | | |
| --- | --- | --- | --- | --- | --- |
| **Variables** | **Overall (N = 995)** | **Excluded (N = 42)** | **Included (N = 953)** | **SMD** | **P-value** |
| **Demographics** |  |  |  |  |  |
| Age, years (Mean ± SD) | 65.56 ± 16.69 | 67.90 ± 12.93 | 65.45 ± 16.84 | 0.163 | 0.242 |
| Female sex, n (%) | 505 (50.8) | 24 (57.1) | 481 (50.5) | 0.134 | 0.397 |
| **Clinical Severity** |  |  |  |  |  |
| SOFA score (Mean ± SD) | 7.23 ± 3.76 | 8.88 ± 4.10 | 7.15 ± 3.73 | 0.441 | 0.01 |
| GCS score (Mean ± SD) | 12.01 ± 3.67 | 11.03 ± 3.27 | 12.05 ± 3.68 | 0.295 | 0.071 |
| **Comorbidities** |  |  |  |  |  |
| Diabetes Mellitus, n (%) | 357 (35.9) | 12 (28.6) | 345 (36.2) | 0.164 | 0.313 |
| Hypertension, n (%) | 548 (55.1) | 19 (45.2) | 529 (55.5) | 0.207 | 0.19 |
| Congestive Heart Failure, n (%) | 215 (21.6) | 11 (26.2) | 204 (21.4) | 0.113 | 0.461 |
| Chronic Kidney Disease, n (%) | 548 (55.1) | 25 (59.5) | 523 (54.9) | 0.094 | 0.554 |
| COPD, n (%) | 180 (18.1) | 6 (14.3) | 174 (18.3) | 0.108 | 0.513 |
| Abbreviations: SMD, standardized mean difference; SOFA, Sequential Organ Failure Assessment; GCS, Glasgow Coma Scale; COPD, chronic obstructive pulmonary disease. | | | | | |
