# Supplementary material for: Elevated blood urea nitrogen-to-albumin ratio is associated with incident sepsis-associated delirium: evidence from a weighted competing-risk analysis in a multicenter cohort study
Source: Front Nutr. 2026 Sep 25;13:1919287. doi: 10.3389/fnut.2026.1919287 (PMC13649452; doi:10.3389/fnut.2026.1919287)
Supplement: Supplementary file 7 [file Table_7.docx]

| Table S5. E-value sensitivity analysis for the association between BAR and 7-day incident delirium | | | | | | |
| --- | --- | --- | --- | --- | --- | --- |
| **Cohort** | **Comparison** | **Weighted 7-day CIF in reference group, % (95% CI)** | **Weighted 7-day CIF in higher-BAR group, % (95% CI)** | **IPTW-adjusted marginal RR (95% CI)** | **E-value for point estimate** | **E-value for CI** |
| **A. Quartile-based analysis (Q4 vs Q1)** | | | | | | |
| MIMIC-IV | BAR Q4 vs Q1 | 42.6 (37.3–47.2) | 55.6 (50.9–59.8) | 1.31 (1.14–1.53) | 1.94 | 1.55 |
| eICU-CRD | BAR Q4 vs Q1 | 19.3 (10.2–34.1) | 37.6 (25.5–49.5) | 1.95 (0.98–3.84) | 3.31 | 1.00 |
| **B. ROC-derived cutoff-based binary analysis** | | | | | | |
| MIMIC-IV | BAR > 6.731 vs BAR ≤ 6.731 | 43.8 (41.2–46.6) | 51.8 (50.3–53.4) | 1.18 (1.10–1.27) | 1.65 | 1.44 |
| eICU-CRD | BAR > 17.2 vs BAR ≤ 17.2 | 28.5 (22.1–34.6) | 40.1 (32.0–48.8) | 1.41 (1.04–1.95) | 2.17 | 1.24 |
| Note. BAR, blood urea nitrogen-to-albumin ratio; CIF, cumulative incidence function; CI, confidence interval; IPTW, inverse probability of treatment weighting; RR, risk ratio. Weighted 7-day cumulative incidences were estimated using the Aalen-Johansen estimator with death treated as a competing event. In analysis A, quartile-based marginal RRs compared the highest BAR quartile (Q4) with the lowest quartile (Q1). In analysis B, cutoff-based marginal RRs compared BAR above versus at/below the cohort-specific ROC-derived candidate cutoff (6.731 in MIMIC-IV and 17.2 in eICU-CRD), selected by the maximum Youden index from the 7-day competing-risk time-dependent ROC analysis. Each contrast used CBPS-derived IPTW estimated for the corresponding exposure parameterization. The 95% CI was obtained using a fixed-weight patient-level percentile bootstrap with 1,000 iterations; all 1,000 bootstrap iterations were valid in all analyses. The E-value for the confidence interval was calculated using the confidence limit closest to the null; when the 95% CI included 1.0, the corresponding E-value was 1.00. | | | | | | |
