# Supplementary material for: Elevated blood urea nitrogen-to-albumin ratio is associated with incident sepsis-associated delirium: evidence from a weighted competing-risk analysis in a multicenter cohort study
Source: Front Nutr. 2026 Sep 25;13:1919287. doi: 10.3389/fnut.2026.1919287 (PMC13649452; doi:10.3389/fnut.2026.1919287)
Supplement: Supplementary file 8 [file Table_8.docx]

| Table S6. Component-specific Fine-Gray associations of BUN, albumin, and BAR with 7-day incident delirium | | | | | | | |
| --- | --- | --- | --- | --- | --- | --- | --- |
| **MIMIC-IV** | | | | | | | |
| **Exposure** | **Variable** | **Model 1 sHR (95% CI)** | **P** | **Model 2 sHR (95% CI)** | **P** | **Model 3 sHR (95% CI)** | **P** |
| BAR | P for trend | - | <0.001 | - | <0.001 | - | 0.002 |
| BAR | Per 1-unit increase | 1.02 (1.01-1.02) | <0.001 | 1.02 (1.01-1.02) | <0.001 | 1.00 (1.00-1.01) | 0.012 |
| BAR | Q2 (vs Q1) | 1.21 (1.02-1.44) | 0.028 | 1.22 (1.02-1.44) | 0.027 | 1.20 (1.02-1.42) | 0.033 |
| BAR | Q3 (vs Q1) | 1.20 (1.01-1.43) | 0.034 | 1.20 (1.02-1.43) | 0.033 | 1.16 (0.98-1.37) | 0.088 |
| BAR | Q4 (vs Q1) | 1.41 (1.16-1.71) | <0.001 | 1.41 (1.16-1.71) | <0.001 | 1.37 (1.14-1.66) | <0.001 |
| BUN | P for trend | - | 0.018 | - | 0.018 | - | 0.090 |
| BUN | Per 1-unit increase | 1.01 (1.00-1.01) | <0.001 | 1.01 (1.00-1.01) | <0.001 | 1.00 (1.00-1.00) | 0.335 |
| BUN | Q2 (vs Q1) | 1.12 (0.93-1.36) | 0.228 | 1.12 (0.93-1.35) | 0.233 | 1.08 (0.90-1.30) | 0.407 |
| BUN | Q3 (vs Q1) | 1.19 (0.99-1.43) | 0.061 | 1.19 (0.99-1.43) | 0.062 | 1.14 (0.95-1.37) | 0.148 |
| BUN | Q4 (vs Q1) | 1.27 (1.03-1.57) | 0.028 | 1.27 (1.03-1.57) | 0.027 | 1.19 (0.96-1.49) | 0.118 |
| Albumin | P for trend | - | <0.001 | - | <0.001 | - | <0.001 |
| Albumin | Per 1-unit increase | 0.87 (0.83-0.91) | <0.001 | 0.87 (0.83-0.91) | <0.001 | 0.85 (0.81-0.89) | <0.001 |
| Albumin | Q2 (vs Q1) | 0.86 (0.75-0.99) | 0.035 | 0.86 (0.75-0.99) | 0.037 | 0.84 (0.72-0.98) | 0.027 |
| Albumin | Q3 (vs Q1) | 0.88 (0.77-1.01) | 0.077 | 0.88 (0.77-1.01) | 0.078 | 0.85 (0.73-0.99) | 0.033 |
| Albumin | Q4 (vs Q1) | 0.68 (0.58-0.79) | <0.001 | 0.68 (0.58-0.79) | <0.001 | 0.70 (0.59-0.83) | <0.001 |

| **eICU-CRD** | | | | | | | |
| --- | --- | --- | --- | --- | --- | --- | --- |
| **Exposure** | **Variable** | **Model 1 sHR (95% CI)** | **P** | **Model 2 sHR (95% CI)** | **P** | **Model 3 sHR (95% CI)** | **P** |
| BAR | P for trend | - | 0.030 | - | 0.030 | - | 0.014 |
| BAR | Per 1-unit increase | 1.01 (1.00-1.02) | 0.007 | 1.01 (1.00-1.02) | 0.015 | 1.01 (1.00-1.02) | 0.073 |
| BAR | Q2 (vs Q1) | 1.37 (0.70-2.68) | 0.355 | 1.38 (0.70-2.69) | 0.348 | 1.35 (0.70-2.60) | 0.366 |
| BAR | Q3 (vs Q1) | 1.53 (0.80-2.92) | 0.199 | 1.54 (0.81-2.95) | 0.190 | 1.46 (0.77-2.80) | 0.248 |
| BAR | Q4 (vs Q1) | 2.08 (1.03-4.17) | 0.040 | 2.09 (1.04-4.20) | 0.039 | 2.11 (1.11-4.02) | 0.023 |
| BUN | P for trend | - | 0.513 | - | 0.542 | - | 0.524 |
| BUN | Per 1-unit increase | 1.01 (1.00-1.01) | 0.003 | 1.01 (1.00-1.01) | 0.011 | 1.01 (1.00-1.01) | 0.054 |
| BUN | Q2 (vs Q1) | 1.10 (0.51-2.38) | 0.806 | 1.10 (0.50-2.40) | 0.809 | 1.05 (0.49-2.24) | 0.899 |
| BUN | Q3 (vs Q1) | 1.55 (0.73-3.29) | 0.256 | 1.55 (0.73-3.27) | 0.253 | 1.55 (0.79-3.03) | 0.203 |
| BUN | Q4 (vs Q1) | 1.13 (0.54-2.37) | 0.738 | 1.12 (0.53-2.36) | 0.770 | 1.10 (0.54-2.25) | 0.795 |
| Albumin | P for trend | - | 0.554 | - | 0.546 | - | 0.488 |
| Albumin | Per 1-unit increase | 0.95 (0.75-1.20) | 0.669 | 0.93 (0.73-1.19) | 0.576 | 0.98 (0.76-1.26) | 0.868 |
| Albumin | Q2 (vs Q1) | 1.16 (0.79-1.72) | 0.445 | 1.16 (0.78-1.71) | 0.459 | 1.14 (0.77-1.68) | 0.512 |
| Albumin | Q3 (vs Q1) | 0.92 (0.61-1.40) | 0.698 | 0.92 (0.60-1.39) | 0.687 | 0.89 (0.59-1.36) | 0.603 |
| Albumin | Q4 (vs Q1) | 0.92 (0.56-1.52) | 0.748 | 0.92 (0.55-1.52) | 0.735 | 0.90 (0.54-1.50) | 0.679 |
| Note. BAR, blood urea nitrogen-to-albumin ratio; BUN, blood urea nitrogen; sHR, subdistribution hazard ratio; CI, confidence interval; CBPS, covariate balancing propensity score; IPTW, inverse probability of treatment weighting. Death before delirium onset was treated as a competing event, and Q1 was the reference category. Quartile and trend analyses used exposure-specific CBPS-derived IPTW; continuous-exposure models did not use quartile-derived IPTW. Model 1, exposure-only model; Model 2, adjusted for age and sex; Model 3, fully adjusted for cohort-specific covariates. | | | | | | | |
