# Supplementary material for: Elevated blood urea nitrogen-to-albumin ratio is associated with incident sepsis-associated delirium: evidence from a weighted competing-risk analysis in a multicenter cohort study
Source: Front Nutr. 2026 Sep 25;13:1919287. doi: 10.3389/fnut.2026.1919287 (PMC13649452; doi:10.3389/fnut.2026.1919287)
Supplement: Supplementary file 9 [file Table_9.docx]

| Table S7. Fine–Gray analyses using cohort-specific ROC-derived BAR cutoffs | | | | | | | |
| --- | --- | --- | --- | --- | --- | --- | --- |
| **Cohort** | **BAR exposure** | **Model 1 sHR (95% CI)** | **P** | **Model 2 sHR (95% CI)** | **P** | **Model 3 sHR (95% CI)** | **P** |
| **MIMIC-IV** | BAR >6.731 vs ≤6.731 | 1.22 (1.11–1.35) | <0.001 | 1.22 (1.11–1.35) | <0.001 | **1.12 (1.01–1.24)** | **0.025** |
|  | Per 1-unit increase | 1.02 (1.01–1.02) | <0.001 | 1.02 (1.01–1.02) | <0.001 | 1.00 (1.00–1.01) | 0.012 |
| **eICU-CRD** | BAR >17.2 vs ≤17.2 | 1.52 (1.09–2.11) | 0.012 | 1.54 (1.11–2.13) | 0.010 | **1.63 (1.19–2.24)** | **0.002** |
|  | Per 1-unit increase | 1.01 (1.00–1.02) | 0.007 | 1.01 (1.00–1.02) | 0.015 | 1.01 (1.00–1.02) | 0.073 |
| Note. BAR, blood urea nitrogen-to-albumin ratio; CI, confidence interval; sHR, subdistribution hazard ratio. Death before delirium onset was treated as a competing event. Cohort-specific candidate BAR cutoffs were identified using the maximum Youden index from 7-day competing-risk time-dependent ROC analyses. Model 1 included the exposure only. Model 2 was adjusted for age and sex. Model 3 was additionally adjusted for cohort-specific covariates. The cutoff analyses used cohort-specific CBPS-derived IPTW. Candidate cutoffs were 6.731 in MIMIC-IV and 17.2 in eICU-CRD. | | | | | | | |
